# Surface-Active Ionic Liquids and Surface-Active Quaternary Ammonium Salts from Synthesis, Characterization to Antimicrobial Properties

**DOI:** 10.3390/molecules29020443

**Published:** 2024-01-16

**Authors:** Marta Wojcieszak, Damian Krystian Kaczmarek, Maciej Karolak, Łukasz Pałkowski, Aneta Lewandowska, Agnieszka Marcinkowska, Katarzyna Dopierała, Katarzyna Materna

**Affiliations:** 1 Faculty of Chemical Technology, Poznan University of Technology, Berdychowo 4, PL-60965 Poznan, Poland; marta.d.wojcieszak@doctorate.put.poznan.pl (M.W.); damian.kaczmarek@put.poznan.pl (D.K.K.); aneta.b.lewandowska@doctorate.put.poznan.pl (A.L.); agnieszka.marcinkowska@put.poznan.pl (A.M.); katarzyna.dopierala@put.poznan.pl (K.D.); 2 Department of Pharmaceutical Technology, Faculty of Pharmacy, Nicolaus Copernicus University, Jurasza 2, PL-85089 Bydgoszcz, Poland; maciej.karolak@cm.umk.pl (M.K.); lukaszpalkowski@cm.umk.pl (Ł.P.)

**Keywords:** surface properties, contact angle, dicamba, antimicrobial activity, leaves surface, model plant membrane

## Abstract

The present work provides new evidence of the ongoing potential of surface-active ionic liquids (SAILs) and surface-active quaternary ammonium salts (surface-active QASs). To achieve this, a series of compounds were synthesized with a yield of ≥85%, and their thermal analyses were studied. Additionally, antimicrobial activity against both human pathogenic and soil microorganisms was investigated. Subsequently, their surface properties were explored with the aim of utilizing SAILs and surface-active QASs as alternatives to commercial amphiphilic compounds. Finally, we analyzed the wettability of the leaves’ surface of plants occurring in agricultural fields at different temperatures (from 5 to 25 °C) and the model plant membrane of leaves. Our results show that the synthesized compounds exhibit higher activity than their commercial analogues such as, i.e., didecyldimethylammonium chloride (DDAC) and dodecyltrimethylammonium bromide (C_12_TAB), for which the CMC values are 2 mM and 15 mM. The effectiveness of the antimicrobial properties of synthesized compounds relies on their hydrophobic nature accompanied by a cut-off effect. Moreover, the best wettability of the leaves’ surface was observed at 25 °C. Our research has yielded valuable insights into the potential effectiveness of SAILs and surface-active QASs as versatile compounds, offering a promising alternative to established antimicrobials and crop protection agents, all the while preserving substantial surface activity.

## 1. Introduction

Many researchers are looking for new and unique compounds with surface properties. Interesting concepts are the surface-active ionic liquids (SAILs) [1,2,3,4,5,6,7] and surface-active quaternary ammonium salts (surface-active QASs) [8,9]. In terms of definition, what these two groups of compounds have in common is the opportunity to possess the properties that distinguish surfactants [3]. Thus, they integrate many aspects such as the ability to wet various surfaces, form a stable foam or reduce surface and interfacial tension [5,10,11,12,13,14]. Well-known structural combinations are based on both monocationic and dicationic compounds with surface activity [5,11,12,15,16]. However, dicationic amphiphilic compounds are increasingly being studied on a larger scale. This is mainly due to the fact that they exhibit higher surface activity, resulting in greater efficiency in lowering the surface tension, a lower Kraft temperature and better solubilization or wettability. In terms of structure, dicationic surface-active compounds are built from two amphiphilic centers connected by a spacer [3]. In their case, the micellization proceeds in an interesting way, which can be determined by the length of the spacer. The unique structure of dicationic compounds impacts not only surface activity but also biological properties [15,17].

A multitude of amphiphilic compounds have been identified as efficient surface-active agents, and researchers have extensively explored their aggregation ability [1,2,3]. The most explored amphiphilic compounds are cationic ones with positively charged ammonium, imidazolium, pyridinium, pyrrolidinium, piperidinium and phosphonium head groups with alkyl chains of different lengths and counter anions with biological properties [3]. Combining a cation with surface activity with an anion with biological properties makes it possible to obtain multifunctional compounds that are being widely studied for potential applications [3,18].

It is noteworthy that, due to the ability to design the structure of multifunctional surface-active compounds, there has been a significant increase in interest in these compounds, especially owing to their use as antimicrobials [19,20,21,22,23]. For example, Shaheen et al. [22] described SAILs in the context of their antimicrobial activity, similar to their quaternary ammonium counterparts. Also, in our recent works, we discussed the role of imidazolium SAILs with high surface activity as a potential tool for destroying microbes [5,19]. Another important use of SAILs and surface-active QASs focuses on sectors such as agriculture. First and foremost, it involves amphiphilic compounds based on (3,6-dichloro-2-methoxy)benzoic acid (dicamba), widely recognized as a selective herbicide. The selectivity of herbicides aims at destroying dicotyledonous plants (weeds) while ensuring the safety of crops [24,25]. It is worth highlighting that the surface properties of compounds with the herbicidal anion make it possible to increase the applicability of a given herbicide by expanding the product’s area of action on the surface of the weed leaf [24,26].

**Figure 1:**
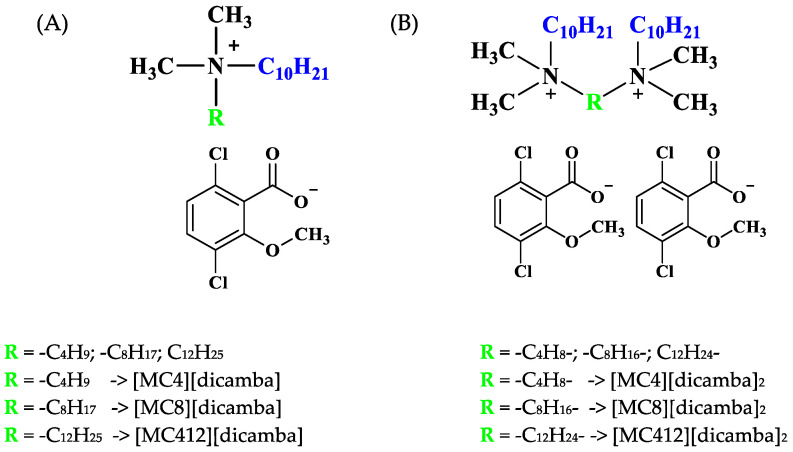
Molecular structure of the monocationic (**A**) and dicationic (**B**) compounds with (3,6-dichloro-2-methoxy)benzoate anion.

The pronounced surface activity reported for certain SAILs and surface-active QASs, coupled with their biological properties and the ever-growing demand for new multifunctional compounds, prompted us to investigate the antimicrobial activity, surface activity and wettability of biological systems of monocationic (a) and dicationic (b) amphiphilic compounds with a (3,6-dichloro-2-methoxy)benzoate anion. Considering that the synthesized compounds are based on (3,6-dichloro-2-methoxy)benzoic acid, we found it reasonable to symbolically designate the anion (3,6-dichloro-2-methoxy)benzoate as dicamba (see Figure 1). We aim to identify prospective antimicrobials and crop protection agents. Building on the direction of our previous study [24], we believe that analyzing the wettability of leaf surfaces of weeds and cereal crops can provide fundamental insights into the compounds’ potential use in agriculture. Conducting wettability tests on leaf surfaces at different temperatures allows us to determine the optimal application temperature. To the best of our knowledge, there are no publications addressing the above research problem. On the other hand, our intention is to broaden the examination by studying the wettability of the prepared model plant membrane of common wheat (*Triticum aestivum* L.) analyzed for the first time in this work. Antimicrobial tests will reveal the extent to which the synthesized compounds resist human pathogenic microorganisms and soil microbes.

## 2. Results and Discussion

### 2.1. Thermal Analysis

The phase transition temperatures of the synthesized monocationic and dicationic compounds were determined from differential scanning calorimetry (DSC) thermograms and are presented in Table 1 and Figure S13. Based on the results obtained, it is concluded that all monocationic compounds can be classified as SAILs, while in the case of dicationic compounds, only **[DC4][dicamba]_2_** is a SAIL while **[DC8][dicamba]_2_** and **[DC12][dicamba]_2_** are surface-active QASs.

For all monocationic SAILs, dicationic SAILs and surface-active QASs, the melting and crystallization temperatures were observed. The **[MC4][dicamba]** with the shortest alkyl chain in the quaternary nitrogen atom substituent and **[DC4][dicamba]_2_** with the four methylene groups in the spacer between two nitrogen atoms exhibited the melting temperature (T_m_) and cold crystallization (T_CC_) during the heating cycle and the crystallization (T_C_) during the cooling cycle_._ The elongation of the alkyl chain from 4 to 12 (CH_2_) groups for SAILs from **[MC4][dicamba]** to **[MC12][dicamba]** caused a decrease in the crystallization temperature, and for SAILs from **[MC4][dicamba]** to **[MC8][dicamba]**, it also caused a decrease in the melting point from 83 °C to 67 °C. Furthermore, the glass transition temperature also decreased. The presence of flexible alkyl chains contributes to the reduction of the melting point, and this effect is caused by the reduced lattice energy [27]. For compounds with shorter alkyl chains, greater attraction between the cation and the anion is observed, while the elongation of the chains reduces the symmetry of the cation and, consequently, reduces the melting point of the investigated compounds. For **[MC12][dicamba],** with the longest alkyl chain in the substituent at the quaternary nitrogen atom, the solid–solid polymorphic transition phase (T_s-s_) was observed. The (T_s-s_) results from conformational changes of the long alkyl chain, causing a change in the density of compounds occurring before the melting point [28]. In the case of the elongation of the alkyl chain of the spacer for dicationic compounds from [**DC4][dicamba]_2_** to **[DC8][dicamba]_2_**, an increase in the melting point from 87 °C to 134 °C was observed. This may be caused by the weakening of electrostatic interactions between the cation and the anion, with the increasing van der Waals interactions beginning to outweigh the symmetry effects [27,29]. However, an additional increase in the number of methylene groups in surface-active QASs from **[DC8][dicamba]_2_** to **[DC12][dicamba]_2_** causes a decrease in the melting point to 121 °C.

The results of the thermal stability of the synthesized compounds are presented in Table 1 and Figure S14. Temperatures corresponding to 5% of mass loss of the sample, determining the beginning of degradation (T_5%_) and for 50% mass loss (T_50%_) were determined. The compounds were characterized by thermal stability in the range of 166 to 187 °C. Thus, for the dicationic compounds, a multistage degradation process was observed compared to monocationic compounds, where for **[DC4][dicamba]_2_**, one main step was observed, for **[DC8][dicamba]_2_**, two steps were observed, and three degradation steps were observed for **[DC12][dicamba]_2_** with the longest alkyl chain (Figure S13). Furthermore, the degradation process of the dicationic SAIL and surface-active QASs starts at higher temperatures (175–187 °C) compared to monocationic SAILs (166–171 °C). Furthermore, monocationic SAILs show similar thermal stability regardless of the increase in the length of the alkyl chain of the substituent at the nitrogen atom. The presented data show that for the dicationic SAIL and surface-active QASs, the thermal stability is dependent on the length of the alkyl chain and increases from 4 to 12 (CH_2_) groups in the spacer between two quaternary nitrogen atoms. The decomposition temperatures at 50% weight loss of the compounds gradually increased with alkyl chain elongation from approximately 212 to 225 °C for monocationic SAILs and from 217 to 263 °C for the dicationic SAIL and surface-active QASs. Such differences may be caused by the progressive fragmentation of the alkyl chain leading to less volatile decomposition products [26].

### 2.2. Self-Aggregation in Water Solution

**Figure 2:**
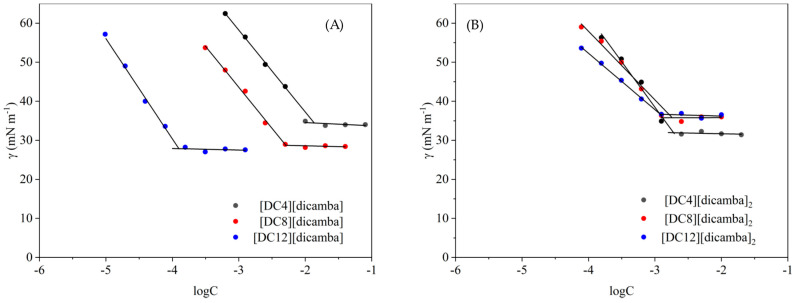
Plot of surface tension (γ) vs. logarithm of concentration (logC) of compounds at 25 °C; (**A**) monocationic and (**B**) dicationic compounds with (3,6-dichloro-2-methoxy)benzoate anion.

In Figure 2, the curves of surface tension (γ) vs. the logarithm concentration of monocationic and dicationic synthesized compounds (logC) at 25 °C are presented (surface tension measurements based on the pendant drop method). The γ of SAIL and surface-active QAS solutions initially decreases with increasing concentration and then finally reaches a plateau. The concentration at the break point in the mentioned plots is taken as the critical micelle concentration (CMC).

The values of parameters such as the critical micelle concentration (CMC), surface tension at the CMC (γ_CMC_), Gibbs free energy of adsorption layer (ΔG^0^_ads_), surface pressure at the CMC (Π_C*MC*_), surface excess concentration at the saturated interface (Γ_max_), minimum surface occupied by a molecule at the interface (A_min_) and adsorption efficiency (pC_20_) of SAILs and surface-active QASs are given in Table 2.

The CMC values for monocationic compounds range from 0.13 to 13.49 mmol L^−1^, and for dicationic compounds, they range from 1.05 to 1.76 mmol L^−1^, respectively. These marked differences can be visually observed in Figure 2 (for monocationic (A) and dicationic compounds (B)). The variation in the molecular structures of synthesized SAILs and surface-active QASs has a significant effect on surface activity, as presented in Table 2. Continuing this thought, the variation in CMC can be connected to the relative hydrophobicity of the alkyl chain or alkyl spacer. Nevertheless, it was established that as hydrophobicity increases by adding four more carbon atoms to the alkyl chain or alkyl spacer, the CMC values of compounds decrease. This observation applies especially to monocationic compounds. Significantly lower results were obtained for dicationic compounds. This might be related to the symmetric structure of this group of compounds, which encourages the formation of micelles and results in low CMC values. A similar suggestion was observed by Saien et al. [14]. Interestingly, **[DC8][dicamba]_2_** exhibited up to seven-fold lower CMC, and **[DC10][dicamba]_2_** showed up to three-fold lower CMC compared to their monocationic analogs. Only **[DC12][dicamba]_2_** showed a propensity to micellize nine-fold higher CMC than **[MC12][dicamba]**. This probably has to do with the length of the spacer, which is crucial for the hydrophobic character responsible for the repulsion between the two head groups.

It is essential not to forget that the number of counterions has an impact on micellization, but this factor is often neglected and insufficiently reported in the literature. This can be attributed to the presence of interactions between aromatic counterions and the amphiphilic part of the structure. Therefore, molecules of amphiphilic compounds tend to make micelle formation more favorable when viewed through an energetic prism [3].

Generally, synthesized SAILs and surface-active QASs showed higher surface activity than conventional surfactants with the same number of carbon atoms in at least one alkyl chain, whereby the CMC values of **DDAC** and **DomphB** were higher only for **[MC4][dicamba]** and **[MC8][dicamba]**. Nevertheless, for compounds **C_10_TAB** and **C_12_TAB**, the CMC values are considerably higher than those of all the synthesized compounds. Moreover, Saien et al. [14] highlighted that obtaining lower CMC values for our compounds is advantageous because it enables their use with lower amounts of reagent compared to conventional surfactants. Moreover, such a situation is desirable not only from an economic point of view but also from an environmental perspective, as lower reagent consumption carries lower costs, and the amount of waste generated is also reduced.

Taking into account the aspects mentioned above and based on the data in Table 2, it is possible to introduce a kind of summary, namely that only a small change in the length of the alkyl chain or spacer, the number of amphiphilic parts and the counter anion can significantly affect the reduction in surface tension and the propensity to micellize [3].

The values of surface tension at the CMC (γ_CMC_) ranged from 28.0 to 34.5 mN m^−1^ (monocationic compounds, see Figure 2A) and from 31.8 to 37.0 mN m^−1^ (dicationic compounds, see Figure 2B). Moreover, Figure 2 shows that the analyzed solutions did not contain impurities because the self-assembly of amphiphilic molecules proceeded without outside interference. Interestingly, lower γ_CMC_ values were obtained for monocationic than for dicationic compounds. This can be explained by stronger interionic interaction in the dicationic amphiphiles, or the second concept concerns the way the molecules of the compound are arranged at the interfacial boundary or their behavior in a bulk of water. A similar trend was observed in our last work and other studies when analyzing monocationic ILs and their dicationic analogs [5,11,12]. The exception in the mentioned tendency was **[MC4][dicamba]**. As highlighted in the literature, monocationic ionic liquids with a short alkyl chain length are distinguished by other physicochemical properties including, i.e., viscosity [4,33,34], which is also seen in our study [5].

According to common knowledge, the adsorption efficiency (pC_20_) and the surface pressure at the CMC (Π_CMC_) are two parameters that reflect the surface activity of compound molecules in an aqueous solution [35]. The adsorption efficiency (pC_20_) for different compounds is in line with the CMC trend. Due to this fact, the elongation of the alkyl chain or alkyl spacer increases the ability to effectively lower the surface tension. However, as expected, Π_CMC_ follows the reverse order to that of γ_CMC_. The ΔG^0^_ads_ values are given in Table 2, and all the values for compounds were negative. This indicates that the adsorption phenomenon occurs spontaneously [36,37]. Moreover, it is observed that with the elongation of the alkyl chain or alkyl spacer, the value of ΔG^0^_ads_ is more negative. Some authors try to find analogies between ΔG^0^_ads_ and the structure of amphiphilic compounds [38,39,40]. For cationic surfactants such as ethyloctadecyldioctylammonium bromide, the value of ΔG^0^_ads_ equals −23.7 kJ mol^−1^ [41], whereas for the non-ionic surfactant 2-[4-(2,4,4-trimethylpentan-2-yl)phenoxy]ethanol (Triton X-100), the value of ΔG^0^_ads_ equals −46.2 kJ mol^−1^ [42]. From the data presented above, it is clear that for fatty alcohols-ethoxylates, the adsorption energy is more negative than for cationic amphiphilic compounds, for which the synthesized compounds are credited.

The maximum surface excess concentration Γ_max_ and the area occupied by a single compound molecule at the air−water interface A_min_ were correlated [43,44]. As the values of A_min_ increase, the values of Γ_max_ decrease, as expected. Wang et al. [45] suggested that a greater value of maximum surface excess concentration or a smaller value of the minimum surface occupied by a molecule at the air/water interface means that the molecules of surface-active compounds at the mentioned interface are more densely and also more tightly packed. Thus, elongation of the alkyl chain or alkyl spacer increases the hydrophobicity of compounds because more molecules exhibit the tendency to approach the air–water interface [46]. However, **[MC12][dicamba]** was an exception to the above rule.

### 2.3. Wettability

**Figure 3:**
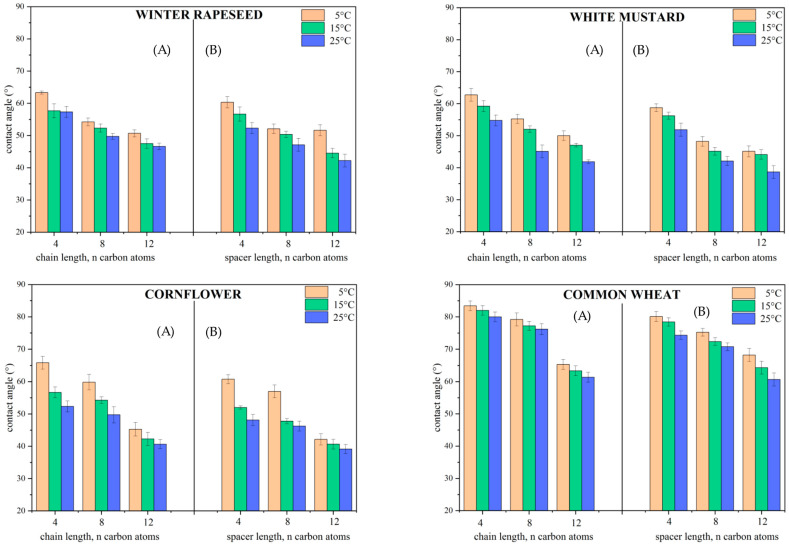
Changes in contact angle values of leaves in relation to temperature; (**A**) monocationic and (**B**) dicationic compounds with (3,6-dichloro-2-methoxy)benzoate anion.

The contact angle (CA) values provide useful information relevant to potential industrial applications [47]. Considering that the tested compounds are structurally composed of an anion with herbicidal activity, it was reasonable to study the wettability of biological surfaces. All CA values are presented in Table S1 and Figure 3. In addition, the CA values resulting from the membrane wettability test are shown in Table S2.

Based on the data in Table S1 and Figure 3, it can be observed that there are no significant differences in the wettability of biological surfaces between monocationic and dicationic compounds. For all studied compounds, the CA values decrease with the elongation of the alkyl chain or alkyl spacer. In our recent works, we extensively described the effect of wettability depending on the surface to be wetted [5,26]. The aforementioned statement is confirmed by this research as well. Continuing this thought, the wettability of the analyzed surfaces can be presented in the following order: cornflower > white mustard > winter rapeseed > common wheat. In this case, the morphological structure of the leaves affects their degree of hydrophobicity, which in turn is reflected in the wettability of the tested surfaces. In contrast, when focusing attention on the effect of temperature on wettability, slightly lower CA values were obtained at 25 °C than at 5 °C (see Figure 3). This trend was described in the literature, where it has been indicated that CA values decrease with increasing temperature [48,49]. Moreover, an interesting relationship, which fits in with the above trend, was observed by Zhang et al. [50], who analyzed the effect of temperature on the wettability of rice leaf surfaces. They suggested that for 25 °C, the CA values were the lowest, consequently attributing the best wettable spreading effect to this phenomenon. Nevertheless, in our study, the maximum difference in CA values between 5 °C and 25 °C did not exceed 21°. This is very promising, as it shows that the compounds act in a similar manner regardless of temperature conditions.

**Figure 4:**
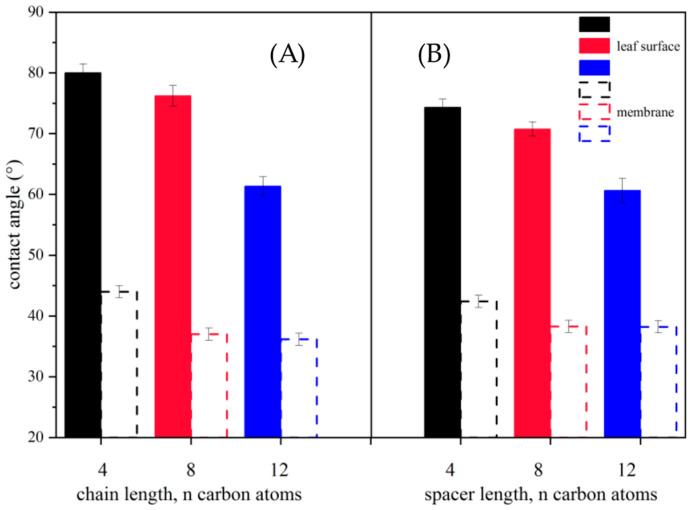
Relationship between values of contact angle obtained for analyzed surfaces. The temperature during the measurements was 25 °C; (**A**) monocationic and (**B**) dicationic compounds with (3,6-dichloro-2-methoxy)benzoate anion.

Concentrating on the surface morphology itself, an attempt was made to compare the values of CA obtained for common wheat leaves and a prepared monolayer that structurally imitates the cell membrane of common wheat leaves (Figure 4).

As shown in Figure 4, there is a significant difference in the wettability of the analyzed surfaces. The smallest difference in CA values was up to 30°, confirming the impact of the morphological structure of these surfaces. Moreover, according to data in the literature [51,52,53], the adaxial side of the leaf includes the cuticle or other biological parts that play a dominant role in wettability. The relationship is of great interest because it illustrates how a drop in amphiphilic compounds with herbicidal activity can behave after penetrating the outer part of the leaf. From our perspective, this research provides new insights into the wettability of biological surfaces, which is so promising that it should be continued in the future.

### 2.4. Antimicrobial Activity

The antimicrobial activity of the analyzed compounds is summarized in Table 3. Additionally, the minimal inhibitory concentration (MIC) values of reference substances commonly used in disinfection and antiseptics, such as didecyldimethylammonium chloride (**DDAC**) and benzalkonium chloride (**BAC**), are presented. It is generally accepted that the antimicrobial activity of this type of compound results from the interaction of the surfactant cation with the cell membrane of the microorganism, leading to membrane disintegration, degradation of proteins and nucleic acids, and cell death. The type of interaction and the mechanism of antimicrobial action depend largely on the concentration of the surfactant, pH, temperature, type of microorganism or the concentration of other ions.

In this study, we determined the impact of newly synthesized compounds on a broad spectrum of microorganisms, namely, Gram-positive, Gram-negative and fungi. We also included *Pseudomonas putida* as a common soil bacterium. It is reported to have some extraordinary properties, such as phytohormone synthesis, nutrient solubilization and excellent root colonization ability [54]. Moreover, it is a well-known soil bacterium used as a standard microorganism to evaluate the ecotoxicity of herbicides. It is of particular interest to determine whether new chemical compounds are able to inhibit pathogenic bacteria while not having a negative effect on soil bacteria.

The dicationic compounds show much higher activity against the tested strains of microorganisms than monocationic compounds. Concerning antimicrobial activity, it can be concluded that dicationic compounds act much more strongly on Gram-positive and fungi than on Gram-negative pathogenic strains. The **[DC12][dicamba]_2_** exhibits the highest activity in this group_._ It is also worth emphasizing that this salt has a higher activity than the standard **DDAC** or **BAC** used. Moreover, dicationic compounds show more effective action against the PPT strain than against Gram-negative pathogenic bacteria. In addition, these compounds are slightly less effective against Gram-positive bacteria and fungi. Therefore, it can be concluded that it is possible to eradicate Gram-positive bacteria without influencing the PPT strain. As compounds with dicamba anions may be used as herbicides, this seems to be promising, as the toxicity of novel dicationic compounds is reduced by taking into account the beneficial PPT strain, which is a part of the soil microflora.

**Figure 5:**
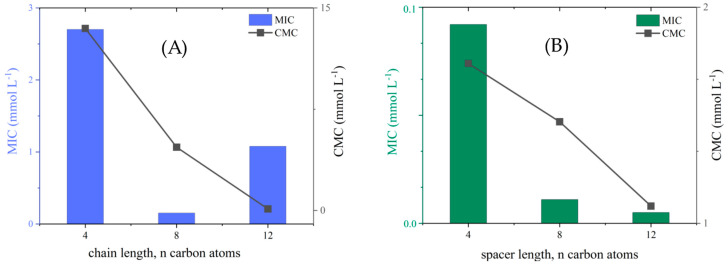
Correlation of MIC vs. CMC values for (**A**) monocationic and (**B**) dicationic compounds with (3,6-dichloro-2-methoxy)benzoate anion.

To better understand the biological activity of the studied compounds, it was decided to correlate their antimicrobial activity with surface properties. Because the above properties have been described in detail in earlier paragraphs, we decided to compare only the MIC values (for Gram-negative pathogenic bacteria, ECO) with the CMC values obtained for all compounds. These results are presented in Figure 5.

Considering the MIC and CMC values, an interesting trend was found for dicationic compounds: as the spacer length increased, both MIC and CMC decreased. This trend was observed for all Gram-negative pathogenic bacteria. However, for the remaining microorganisms, a clear relationship could not be formulated. For monocationic compounds, a parabolic dependence of the biological activity on the CMC values and the elongation of the alkyl chain was observed. This phenomenon, known as the cut-off effect, is widely described in the literature [5,21,55]. It should be noted that this trend was found for Gram-negative pathogenic bacteria and soil microorganisms. The optimum antimicrobial activity was reported for **[MC8][dicamba]**. This monocationic SAIL displayed the best antimicrobial properties with a similar propensity to micellization as surfactants.

## 3. Materials and Methods

### 3.1. Materials

Decyldimethylamine (90%, CAS number: 1120-24-7), 1,4-dibromobutane (98%, CAS number: 110-52-1), 1,8-dibromooctane (98%, CAS number: 4549-32-0), 1,12-dibromododecane (98%, CAS number: 3344-70-5), 1-bromobutane (99%, CAS number: 109-65-9), 1-bromooctane (99%, CAS number: 111-83-1), 1-bromododecane (97%, CAS number: 143-15-7), potassium hydroxide (90%, CAS number: 1310-58-3), acetonitrile (99%, CAS number: 75-05-8), ethyl acetate (99%, CAS number: 141-78-6), hexane (95%, CAS number: 110-54-3), methanol (99%, CAS number: 100-72-1) and acetone (99%, CAS number: 67-64-1) were purchased from Sigma-Aldrich and used without further purification. (3,6-dichloro-2-methoxy)benzoic acid (95%, CAS number: 1918-00-9) was purchased from Pol-Aura and used without further purification.

### 3.2. Synthesis

#### 3.2.1. Synthesis of Alkyldecyldimethylammonium Bromide

**Figure 6:**
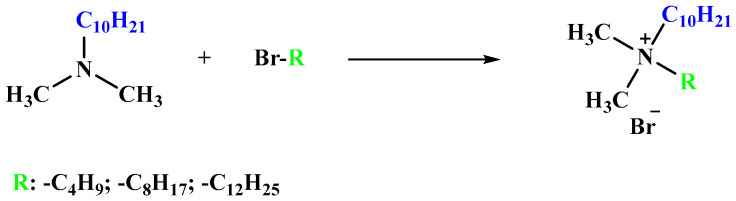
The reaction pathway followed for the synthesis of alkyldecyldimethylammonium bromide.

Decyldimethylamine (0.05 mol) and acetonitrile (80 cm^3^) were placed in a reactor containing a magnetic stirring bar. Then, a stoichiometric amount of 1-bromobutane, 1-bromooctane or 1-bromododecane was added. The reaction was conducted for 24 h at 80 °C, after which the remaining substrates were extracted from acetonitrile by washing three times with hexane (50 cm^3^). Next, the solvent was removed using a rotary evaporator, and the obtained products were dried under reduced pressure at 70 °C for 24 h. In Figure 6, a scheme for the synthesis of alkyldecyldimethylammonium bromide is presented.

#### 3.2.2. Synthesis of Alkane-1,ω-bis(Decyldimethylammonium) Dibromide

**Figure 7:**
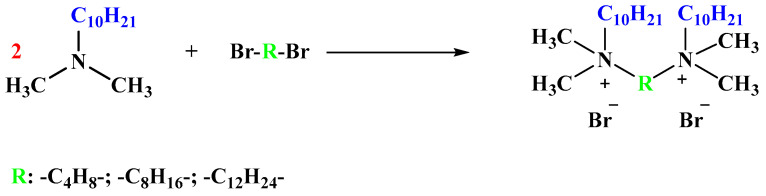
The reaction pathway followed for the synthesis of alkane-1,ω-bis(decyldimethylammonium) dibromide.

In a round-bottomed flask containing a magnetic stirring bar, 1,4-dibromobutane, 1,8-dibromooctane or 1,12-dibromododecane (0.05 mol) was dissolved in 50 mL of acetonitrile, followed by the addition of 0.1 mol of decyldimethylamine dissolved in 25 mL of acetonitrile. The reaction was conducted at the boiling point temperature of the solvent for 24 h. Then, the solvent was evaporated, and 100 mL of ethyl acetate was added. The product precipitated in the form of a white solid and was isolated by filtration (washed with small portions of ethyl acetate). After filtration, the compound was dried under vacuum for 24 h at 70 °C. In Figure 7, a scheme for the synthesis of alkane-1,ω-bis(decyldimethylammonium) dibromide is presented.

#### 3.2.3. Metathesis Reaction

**Figure 8:**
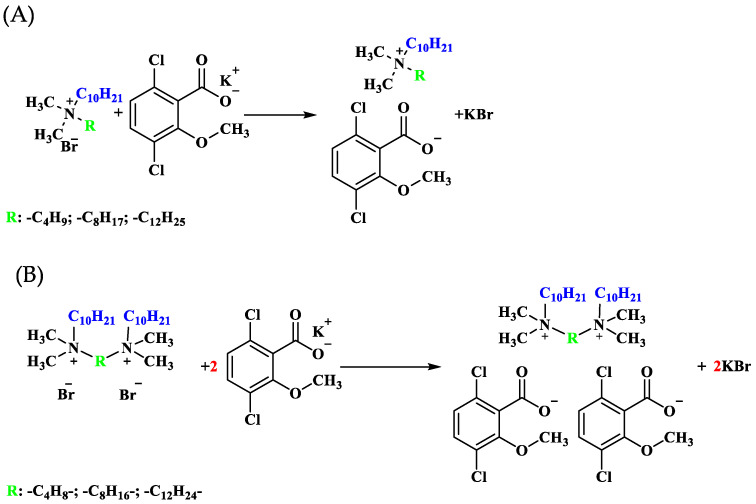
Reaction pathway followed for the synthesis of monocationic (**A**) and dicationic (**B**) compounds with (3,6-dichloro-2-methoxy)benzoate anion.

In a round-bottomed flask equipped with a magnetic stirring bar, 0.05 mol of ammonium bromide or bis-ammonium dibromide was dissolved in 25 mL of methanol. Subsequently, 0.05 or 0.1 mol of the potassium salts of (3,6-dichloro-2-methoxy)benzoic acid (dicamba) were added to the solution, followed by stirring for 0.5 h at room temperature. The solvent was then evaporated using a rotary evaporator, and the resulting products were dissolved in 50 mL of anhydrous acetone. The precipitate (byproduct) obtained was separated via vacuum filtration, and the solvent was evaporated. The final product was dried under vacuum at 50 °C for 24 h. The spectra of the compounds are described in the Supplementary Data (Figures S1–S12). Moreover, the schemes of the synthesis of compounds with the (3,6-dichloro-2-methoxy)benzoate anion are presented in Figure 8.

The yields for SAILs and surface-active QASs were obtained, and their precursors (alkyldecyldimethylammonium bromides and alkane-1,ω-bis(decyldimethylammonium) dibromides) are presented in Table 4.

### 3.3. Thermal Analysis

Thermogravimetric investigations (TGA) were used to study the thermal stability of the synthesized compounds. Measurements were performed on a TG 209 F3 Tarsus analyzer (NETZSCH-Geratebau GmbH, Selb, Germany). Approximately 10 mg of the sample was placed in a platinum crucible and then analyzed in the temperature range from 30 to 600 °C with a heating rate of 10 °C min^−1^ under a nitrogen atmosphere (flow of purge gas 20 mL min^−1^ and protective gas 10 mL min^−1^).

The thermal transition temperature was determined by the differential scanning calorimetry (DSC) method using a DSC1 instrument (Mettler-Toledo, Greifensee, Switzerland). Measurements were carried out in an argon atmosphere (flow rate of 50 mL min^−1^) with a heating rate of 10 °C min^−1^ in a temperature range from 30 to 160 °C. The melting temperature (T_m_), glass transition temperature (T_g_), cold crystallization temperature (T_cc_) and solid–solid transition temperature (T_s-s_) were determined from the heating cycle of the DSC thermograms. Crystallization temperatures (T_c_) were determined from the cooling cycles in DSC traces.

### 3.4. Self-Aggregation in Water Solution

Surface activity was measured using a DSA 100 analyzer (Krűss, Hamburg, Germany) and controlled by constant temperature (Fisherbrand FBH604 thermostatic bath; Fisher, Schwert, Germany). During the measurements, a photo of the drop was taken and digitized by a CCD camera. The surface tension (γ in mN m^−1^) was determined by examining the drop profile based on Laplace’s mathematical equation. Parameters such as the critical micelle concentration (CMC), surface tension at the CMC (γ_CMC_), Gibbs free energy of adsorption layer (ΔG^0^_ads_), surface pressure at the CMC (Π*_CMC_*), surface excess concentration at the saturated interface (Γ_max_), minimum surface occupied by a molecule at the interface (A_min_) and adsorption efficiency (pC_20_) were determined. The formulas (which are collected in the Supplementary Data) used to determine the values of the various parameters were also described in previous works [16,24]. Surface tension measurements for each concentration were conducted until they stabilized (the number of unit measurements was not less than 360). The standard deviation of the results obtained from the measurements was ±0.1 mN m^−1^, and the uncertainty was no greater than 0.9%.

### 3.5. Wettability

Wettability is based on contact angle (CA) measurements. The CA values were determined at different temperatures (5, 15 and 25 °C). They rely on the study of a drop of spray solution deposited on a biological surface. After correctly identifying the drop’s shape and the contact line, the drop’s shape is fitted to the property mathematical model. In this study, the biological systems of common wheat (*Triticum aestivum* L.), cornflower (*Centaurea cyanus* L.), winter rapeseed (*Brassica napus* L.) and white mustard (*Sinapis alba* L.) were analyzed as the solid phase. Biological systems were performed on the adaxial side of the leaves. The leaves of biological systems were trimmed immediately prior to the measurement. Subsequently, they were affixed to the table within a temperature-controlled chamber (5 or 15 or 25 °C, respectively). The part of the leaf sticking out of the table was then cut off with a scalpel. The leaf surface was not touched during the tests to avoid disturbing the morphology of the leaves, especially the surface waxes. Following the establishment of stable conditions within the chamber, 2.0 μL droplets were dispensed using a 1 mL syringe equipped with a needle measuring 0.5 mm in diameter. Measurement results were recorded until the contact angle value stabilized, but not for less than 120 s. The measurement was repeated nine times, each time on a new sample (plant leaf). Additionally, contact angle measurements were conducted on the membrane surface (for the method of preparation, please refer to Section 3.7). Contact angle tests on the membrane were performed analogously to those on the leaves’ surface.

### 3.6. Statistical Analysis

The standard error of the mean (SEM) method is defined by the estimated standard errors of the mean of which values were calculated on the basis of the equation below:

$$SEM=\frac{s}{n^{0.5}}\;$$

SEM is the standard error of the mean,

s is sample standard deviation,

n is the number of replications.

One-way analysis of variance (ANOVA) at α = 0.05 was conducted to determine the significance of the obtained results.

### 3.7. Preparation of Membrane

The model plant membrane was prepared using the Langmuir–Blodgett (LB) method. The Teflon trough with a surface area of 273 cm^2^ (KSV Nima, Helsinki, Finland) was filled with ultrapure water (18 MΩ·cm, pH 6.20, TOC 1−3 ppb). The plant lipids 1-palmitoyl-2-oleoyl-glycero-3-phosphocholine (POPC), 1,2-dipalmitoyl-sn-glycero-3-phosphoethanolamine (DPPE), sodium salt of 1,2-dioleoyl-sn-glycero-3-phospho-(1’-rac-glycerol) (DOPG-Na), stigmasterol (STIG) and β-sitosterol (β-SIT) were dissolved in chloroform (Uvasol, Merck, Rahway, NJ, USA). The total concentration was 1 mg/mL, and the ratio of components in the POPC/DPPE/DOPG-Na/STIG/β-SIT mixture was 1:1:1:2:2. The mixture of lipids was spread at the air–water interface dropwise using a gas-tight syringe. After evaporation of the solvent, the lipid molecules were symmetrically compressed using the barriers moving at a rate of 10 mm min^−1^ until the condensed film was obtained, i.e., the surface pressure reached 35 mN m^−1^. Then, the transfer of the film onto the solid substrate was initiated. The monolayer was deposited on the microscopic glass slide during a single upstroke controlled by the automated dipper and the software (KSV Nima, Helsinki, Finland). The deposition rate was 2 mm min^−1^. All transfers were characterized by a high transfer ratio (TR) determined as the ratio between the decrease in the monolayer area during a deposition stroke and the area of the glass slide.

### 3.8. Antimicrobial Activity

The compounds under analysis were assessed for their antimicrobial potential against various strains including *Staphylococcus aureus* ATCC 25,213 (SAU), *Pseudomonas aeruginosa* ATCC 27,853 (PAE), *Klebsiella pneumoniae* ATCC 700,603 (KPN), *Escherichia coli* ATCC 25,922 (ECO), *Enterococcus faecalis* ATCC 29,212 (EFA), *Candida albicans* ATCC 90,028 (CAL) and *Pseudomonas putida* ATCC 49,128 (PPT). These strains were obtained from Microbiologics in San Diego, CA, USA. To create bacterial and fungal suspensions with the equivalent turbidity of 0.5 McFarland standards, growth from blood trypticase soy agar (TSA, BBL) plates was suspended in 2 mL of sterile saline. The antibacterial activity of the compounds was gauged using the disk-diffusion method, determining the minimal inhibitory concentration (MIC, % *w*/*v*) values ranging from 1.0 to 0.000125. Two controls were utilized in this experiment: The growth control involved a bacterial suspension devoid of antimicrobial agents, facilitating the observation of bacterial growth without any interference. The sterile control solely contained a growth medium without test bacteria or antibacterial agents, expected to display no bacterial growth after incubation, thus establishing a baseline for comparison. Mueller–Hinton agar (bio-Merieux, Lombard, IL, USA) served as the test medium. MIC was defined as the lowest concentration at which no visible bacterial growth was observed, except for a barely visible haze, which was disregarded. MIC readings were taken at 37 °C after 24 or 48 h of culture for different bacteria and Candida spp. The agar dilution procedure for testing microorganisms aligns with the Clinical and Laboratory Standards Institute guidelines [56].

## 4. Conclusions

In this work, a series of compounds, including SAILs and surface-active QASs, were synthesized and analyzed. These amphiphilic compounds exhibit a tendency to reduce the surface tension to 28.0–37.0 mN m^−1^, behaving as cationic surfactants. The surface activity of all compounds manifests in an increase in micellization tendency with the elongation of the spacer or alkyl chain. It is worth emphasizing that the crucial driving force of the micellization of the studied compounds is related to the hydrophobic interaction between their alkyl parts.

Our investigation demonstrates that the highest contrast in CA values between 5 °C and 25 °C did not surpass 21°. This is an encouraging finding, indicating that the compounds exhibit consistent behavior irrespective of temperature variations. **[MC12][dicamba]** and **[DC12][dicamba]_2_** are the best potential wetting agents for leaf surfaces. This was suggested by the lowest CA values regardless of the temperature conditions prevailing during the measurements. The efficiency in wettability of biological surfaces for all synthesized compounds increases with the elongation of the alkyl chain connected to the polar head group or with the lengthening of the spacer connecting the two hydrophobic centers. Moreover, the results obtained from the study of membrane surface wettability are very interesting because they apparently show how wettability is affected by the morphological structure of the leaf. In addition, they give new undescribed light for further research, especially important from the point of view of the potential use of the synthesized compounds as crop protection agents.

Antimicrobial activity increases with the increase in the number of carbon atoms in the alkyl chain or spacer. Dicationic compounds show much higher activity against tested strains of microorganisms compared to monocationic ones. The relationship between MIC and CMC confirms that CMC is definitely an ideal indicator of hydrophobicity that can be used for microbial control. For SAILs, the cutoff effect can be seen, which is described in the literature [5,21,57]. The degree to which SAILs are prone to micellization probably limits the rate of diffusion to the microbial surface and the ability to permeabilize the cell membrane. However, antimicrobial activity does not depend only on the amphiphilic structure of the chemical compound interacting with the cell membrane but also on the structure itself (Gram-positive and Gram-negative bacteria).

This article can contribute to a more comprehensive understanding of monocationic and dicationic amphiphilic compounds (based on SAILs and surface-active QASs) and underline the exceptionality, primarily as replacements for commercial amphiphilic compounds but also as antimicrobials and crop protection agents. Therefore, an attempt was made to determine the structure of the compounds, which definitely affects the micellization and biological activity of the compounds described in this study. Detailed knowledge of the interface phenomena of newly formed SAILs and surface-active QASs is important for understanding their environmental fate, which is fundamental to the philosophy of green chemistry.

## Figures and Tables

**Table 1:** Thermal properties of synthesized compounds (*T*_g_—glass transition temperature, *T*_cc_—cold crystallization temperature, *T*_s−s_—solid–solid transition temperature, *T*_m_—melting temperature, *T*_c_—crystallization temperature, *T*_5%_—the temperature of mass loss of 5% of the sample, *T*_50%_—the temperature of mass loss of 50% of the sample).

Abbreviation	T_cc1_ (°C)	T_c1_ (°C)	T_c2_ (°C)	T_s-s1_ (°C)	T_s-s2_ (°C)	T_m_ (°C)	T_g_ (°C)	T_5%_ (°C)	T_50%_ (°C)
**[MC4][dicamba]**	61.00	66.17	-	-	-	83.83	−39.52	170.5	212.4
**[MC8][dicamba]**	-	38.83	-	-	-	67.33	−59.20	165.5	215.5
**[MC12][dicamba]**	-	10.33	65.00	28.83	46.17	68.83	−51.87	168.0	224.7
**[DC4][dicamba]_2_**	53.33	75.33	-	-	-	86.83	−2.53	175.4	216.9
**[DC8][dicamba]_2_**	-	73.33	-	-	-	134.30	−10.04	177.0	243.1
**[DC12][dicamba]_2_**	-	49.33	-	-	-	121.50	-	186.6	263.0

**Table 2:** Surface active parameters for studied compounds.

Abbreviation	CMC (mmol L^−1^)	pC_20_	γ_CMC_ (mN m^−1^)	Π_CMC_ (mN m^−1^)	Γ_max_ × 10^6^ (mol m^−2^)	A_min_ × 10^19^ (m^2^)	ΔG^0^_ads_ (kJ mol^−1^)
**[MC4][dicamba]**	13.49	2.71	34.5	38.3	4.95	3.35	−18.74
**[MC8][dicamba]**	4.70	3.39	28.8	44.0	3.93	4.22	−24.12
**[MC12][dicamba]**	0.13	4.86	28.0	44.8	4.52	3.67	−30.87
**[DC4][dicamba]_2_**	1.76	3.54	31.8	41.0	4.81	3.46	−23.88
**[DC8][dicamba]_2_**	1.48	3.66	35.9	36.9	2.98	5.57	−27.46
**[DC12][dicamba]_2_**	1.05	3.99	37.0	35.8	2.67	6.23	−30.00
**DDAC**	2.00 ^a^						
**DomphB**	1.78 ^b^						
**C_10_TAB**	67.00 ^c^						
**C_12_TAB**	15.00 ^c^						

**DDAC**—didecyldimethylammonium chloride, **DomphB**—domiphen bromide, **C_10_TAB**—decyltrimethylammonium bromide, **C_12_TAB**—dodecyltrimethylammonium bromide. γ_0_ surface tension of water, 72.8 mN m^−1^ under measurement conditions. ^a^ Ref. [30], ^b^ Ref. [31], ^c^ Ref. [32].

**Table 3:** MIC values for studied compounds.

MIC (mmol L^−1^)							
	Human Pathogenic Microorganisms						Soil Microorganism
	G(+)		G(−)			Fungi	G(−)
Abbreviation	SAU	EFA	PAE	ECO	KPN	CAL	PPT
**[MC4][dicamba]**	0.173	0.346	10.811	2.703	10.811	0.670	2.703
**[MC8][dicamba]**	0.019	0.039	1.196	0.154	0.309	0.039	0.077
**[MC12][dicamba]**	0.002	0.004	4.350	1.079	1.079	0.004	1.079
**[DC4][dicamba]_2_**	0.006	0.003	0.715	0.092	1.442	0.006	0.023
**[DC8][dicamba]_2_**	0.011	0.011	0.336	0.011	0.087	0.011	0.087
**[DC12][dicamba]_2_**	0.003	0.003	0.082	0.005	0.041	0.003	0.041
**DDAC**	0.004	0.007	0.856	0.014	0.856	0.007	0.442
**BAC**	0.028	0.014	1.765	0.057	0.456	0.014	0.459

SAU—*Staphylococcus aureus* ATCC 25213, PAE—*Pseudomonas aeruginosa* ATCC 27853; KPN—*Klebsiella pneumoniae* ATCC 700603, ECO—*Escherichia coli* ATCC 25922, EFA—*Enterococcus faecalis* ATCC 29212, CAL—*Candida albicans* ATCC 90028 and PPT—*Pseudomonas putida* ATCC 49128.

**Table 4:** Synthesized compounds.

**Abbreviation**	**-R**	**Anion**	**Yield (%)**	**Abbreviation**	**-R-**	**Anion**	**Yield (%)**
**[MC4][Br]**	-C_4_H_9_	Br	90	**[DC4][Br]_2_**	-C_4_H_8_-	Br	89
**[MC8][Br]**	-C_8_H_17_		85	**[DC8][Br]_2_**	-C_8_H_16_-		90
**[MC12][Br]**	-C_12_H_25_		89	**[DC12][Br]_2_**	-C_12_H_24_-		91
**Abbreviation**	**-R**	**Anion**	**Yield (%)**	**Abbreviation**	**-R-**	**Anion**	**Yield (%)**
**[MC4][dicamba]**	-C_4_H_9_	Dicamba	95	**[DC4][dicamba]_2_**	-C_4_H_8_-	Dicamba	92
**[MC8][dicamba]**	-C_8_H_17_		96	**[DC8][dicamba]_2_**	-C_8_H_16_-		90
**[MC12][dicamba]**	-C_12_H_25_		94	**[DC12][dicamba]_2_**	-C_12_H_24_-		89

## Data Availability

Data generated and analyzed during this study are included in this manuscript and Supplementary Materials.

## Supplementary Materials

The following supporting information can be downloaded at https://www.mdpi.com/article/10.3390/molecules29020443/s1, Figure S1: 1H NMR spectrum of butyldecyldimethylammonium (3,6-dichloro-2-methoxy)benzoate (**1**); Figure S2: 13C NMR spectrum of butyldecyldimethylammonium (3,6-dichloro-2-methoxy)benzoate (**1**); Figure S3: ^1^H NMR spectrum of octyldecyldimethylammonium (3,6-dichloro-2-methoxy)benzoate (**2**); Figure S4: ^13^C NMR spectrum of octyldecyldimethylammonium (3,6-dichloro-2-methoxy)benzoate (**2**); Figure S5: ^1^H NMR spectrum of dodecyldecyldimethylammonium (3,6-dichloro-2-methoxy)benzoate (**3**); Figure S6: ^13^C NMR spectrum of dodecyldecyldimethylammonium (3,6-dichloro-2-methoxy)benzoate (**3**); Figure S7: ^1^H NMR spectrum of tetramethylene-1,4-(decyldimethylammonium) di[(3,6-dichloro-2-methoxy)benzoate] (**4**); Figure S8: ^13^C NMR spectrum of tetramethylene-1,4-(decyldimethylammonium) di[(3,6-dichloro-2-methoxy)benzoate] (**4**); Figure S9: ^1^H NMR spectrum of octamethylene-1,8-bis(decyldimethylammonium) di[(3,6-dichloro-2-methoxy)benzoate] (**5**); Figure S10: ^13^C NMR spectrum of octamethylene-1,8-bis(decyldimethylammonium) di[(3,6-dichloro-2-methoxy)benzoate] (**5**); Figure S11: ^1^H NMR spectrum of dodecamethylene-1,12-bis(decyldimethylammonium) di[(3,6-dichloro-2-methoxy)benzoate] (**6**); Figure S12: ^13^C NMR spectrum of dodecamethylene-1,12-bis(decyldimethylammonium) di[(3,6-dichloro-2-methoxy)benzoate] (**6**); Figure S13: DSC thermograms of the monocationic (A) and dicationic (B) compounds with (3,6 dichloro-2-methoxy)benzoate anion; Figure S14: (A) TG and (B) DTG curves for investigated compounds. Table S1: Contact angle values of compounds (biological surface); Table S2: Contact angle values of compounds (membrane).
