# Supplementary material for: Surface-Active Ionic Liquids and Surface-Active Quaternary Ammonium Salts from Synthesis, Characterization to Antimicrobial Properties
Source: Molecules. 2024 Jan 16;29(2):443. doi: 10.3390/molecules29020443 (PMC10819711; doi:10.3390/molecules29020443)
Supplement: Supplementary file 1 [file molecules-29-00443-s001.zip › molecules-2764458-supplementary.pdf]

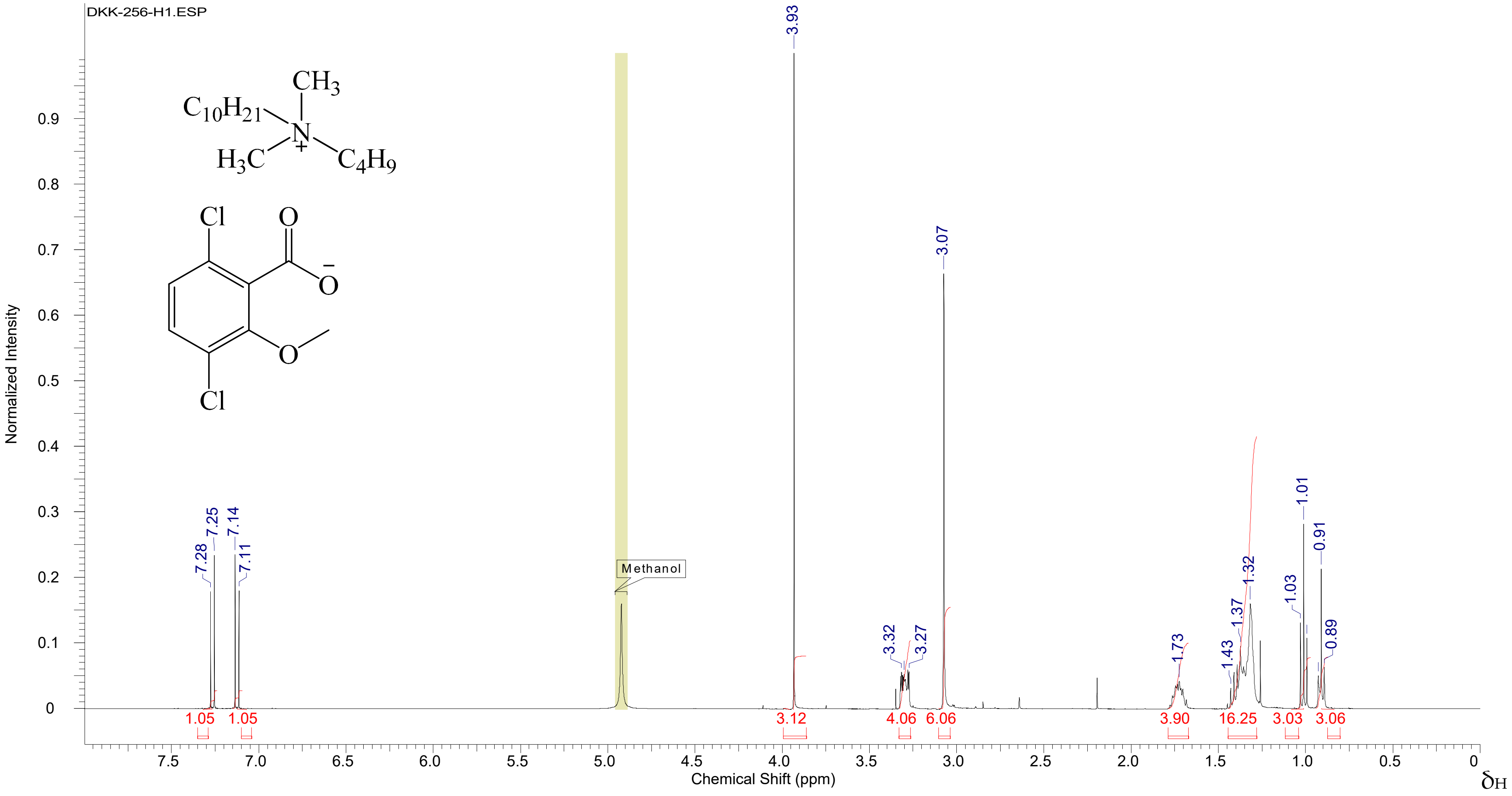

(METHANOL-d4): 7.28 (1H, d, J=9 Hz), 7.14 (1H, d, J=9 Hz), 3.93 (3H, s), 3.27 - 3.32 (4H, m), 3.07 (6H, s), 1.73 (4H, m), 1.32 - 1.43 (16H, m), 0.99 - 1.03 (3H, m), 0.89 - 0.93 (3H, m).

Figure S1. ^{1}H NMR spectrum of butyldecyldimethylammonium (3,6-dichloro-2-methoxy)benzoate (**1**)

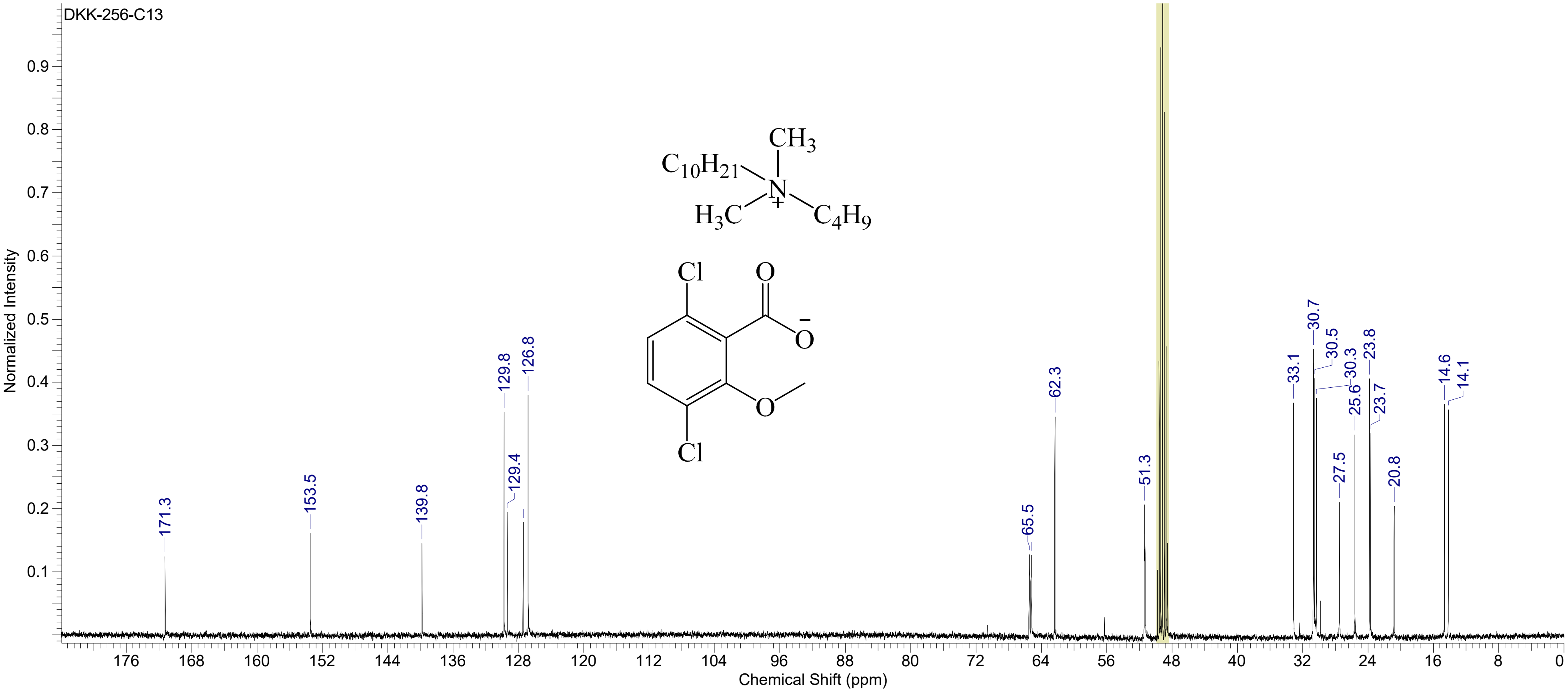

δC (METHANOL-d4): 171.3, 153.5, 139.8, 129.8, 129.4, 127.4, 126.8, 65.5, 65.2, 62.3, 51.3 (2C), 33.1, 30.7, 30.7, 30.5, 30.3, 27.5, 25.6, 23.8, 23.7, 20.8, 14.6, 14.1.

Figure S2. ^{13}C NMR spectrum of butyldecyldimethylammonium (3,6-dichloro-2-methoxy)benzoate (**1**)

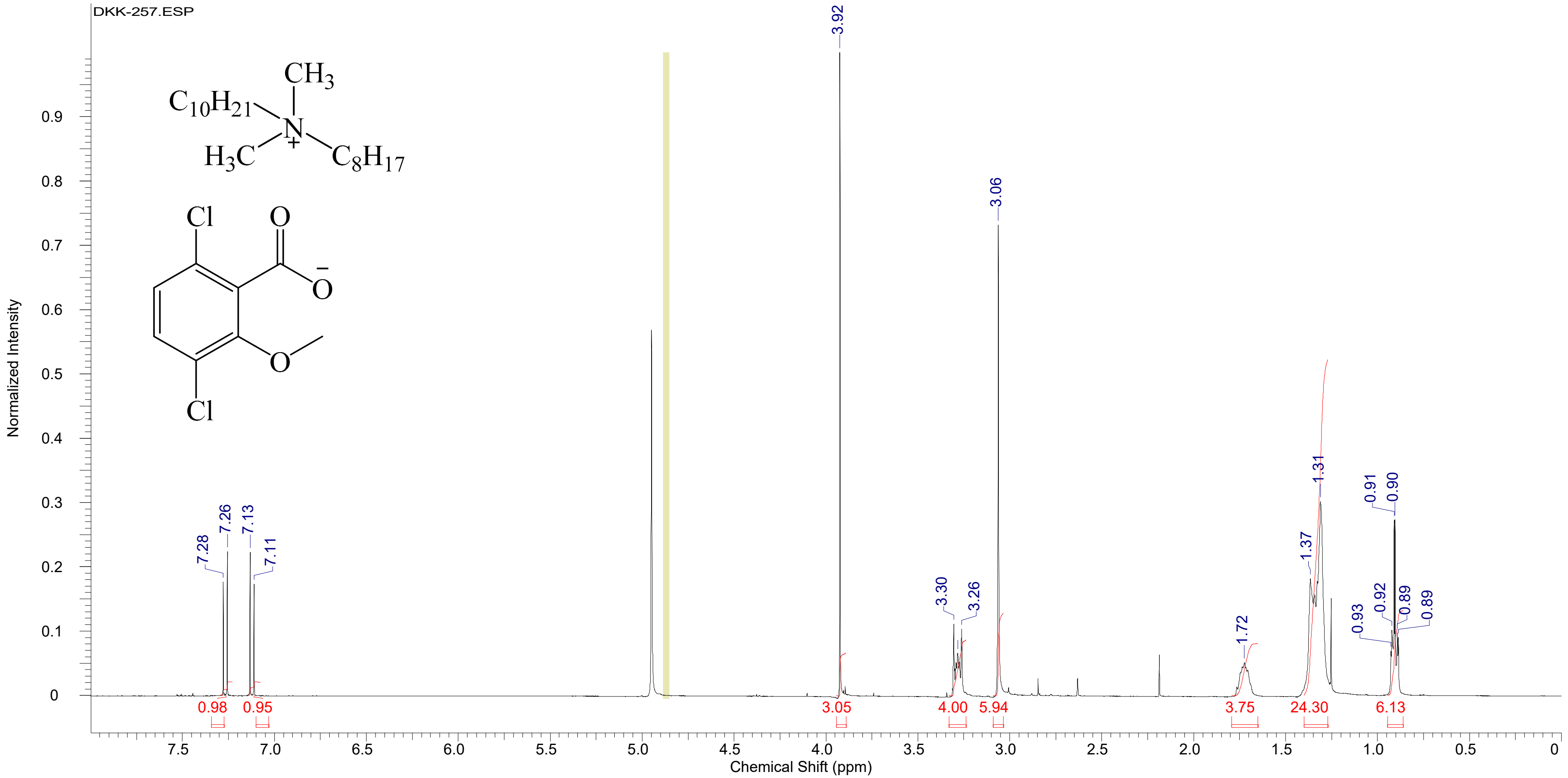

δ_H (METHANOL-d4): 7.28 (1H, d, J=9 Hz), 7.13 (1H, d, J=9 Hz), 3.92 (3H, s), 3.26 - 3.30 (4H, m), 3.06 (6H, s), 1.72 (4H, m), 1.31 - 1.37 (24H, m), 0.89 - 0.93 (6H, m).

Figure S3. ^{1}H NMR spectrum of octyldecyldimethylammonium (3,6-dichloro-2-methoxy)benzoate (**2**)

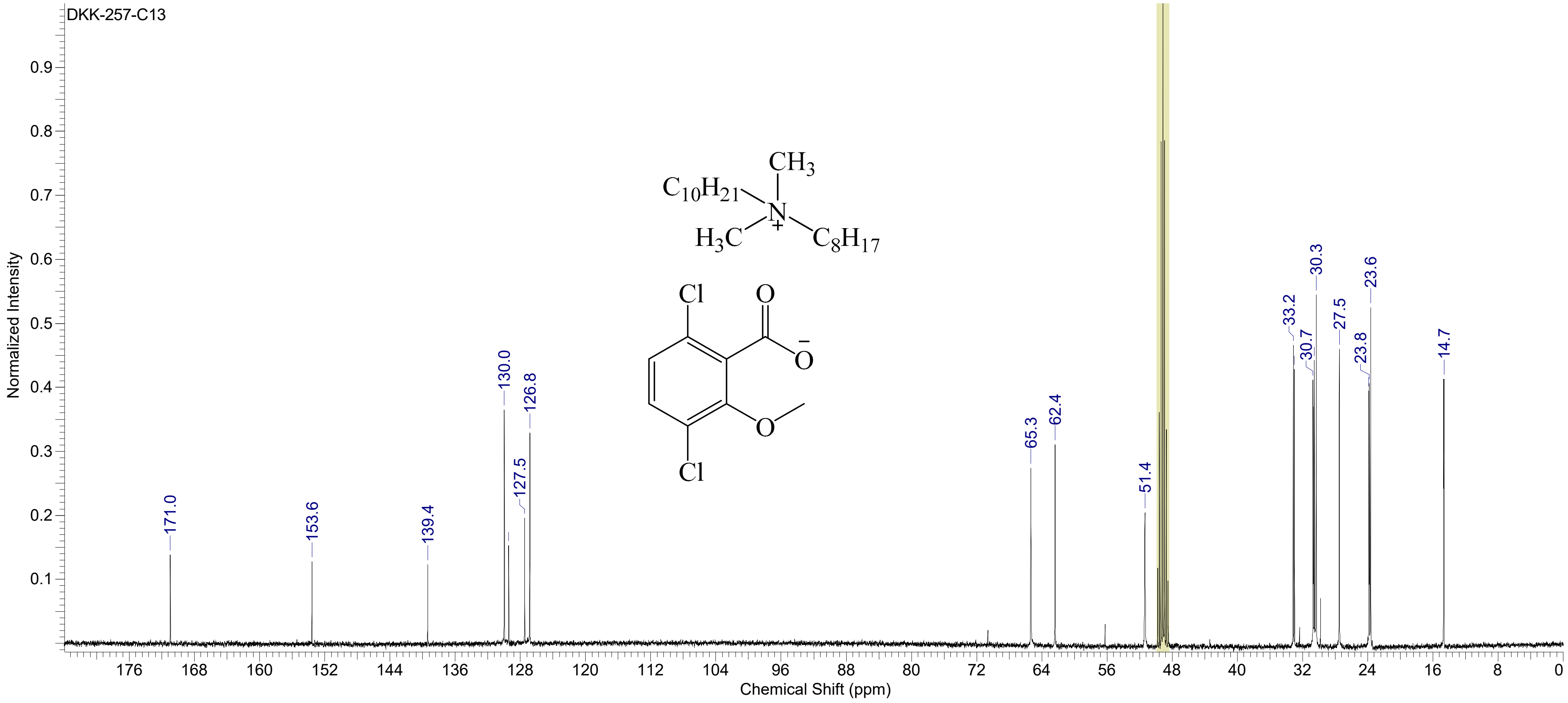

δC (METHANOL-d4): 171.0, 153.6, 139.4, 130.0, 129.4, 127.5, 126.8, 65.3 (2C), 62.4, 51.3 (2C), 33.2 (2C), 30.7, 30.5, 30.3 (2C), 30.3 (2C), 27.5 (2C), 23.8 (2C), 23.6 (2C), 14.7 (2C).

Figure S4. ^{13}C NMR spectrum of octyldecyldimethylammonium (3,6-dichloro-2-methoxy)benzoate (**2**)

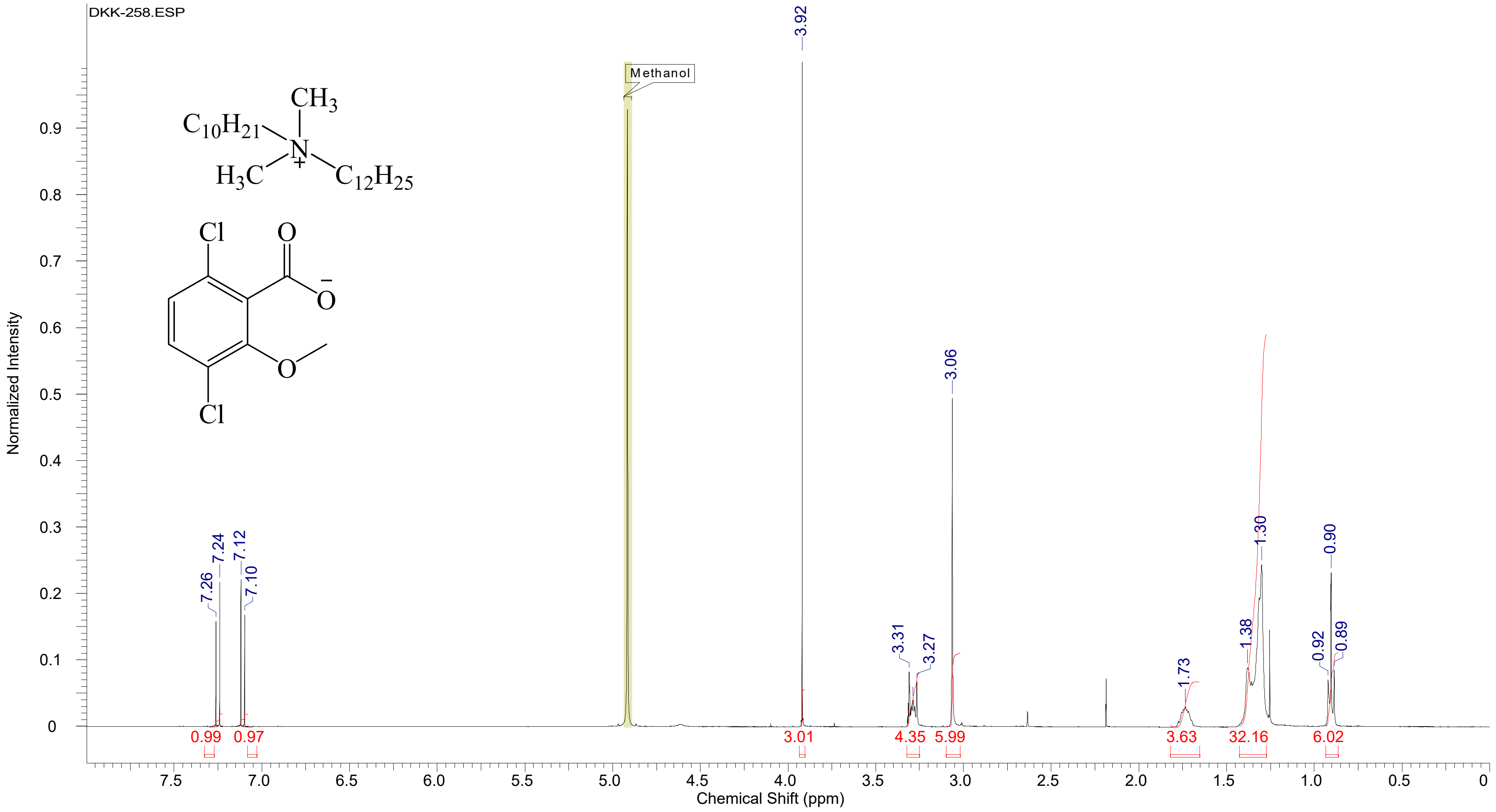

δH (METHANOL-d4): 7.26 (1H, d, J=9 Hz), 7.12 (1H, d, J=9 Hz), 3.92 (3H, s), 3.27 - 3.31 (4H, m), 3.06 (6H, s), 1.73 (4H, m), 1.30 - 1.38 (32H, m), 0.89 - 0.92 (6H, m).

Figure S5. ^{1}H NMR spectrum of dodecyldecyldimethylammonium (3,6-dichloro-2-methoxy)benzoate (**3**)

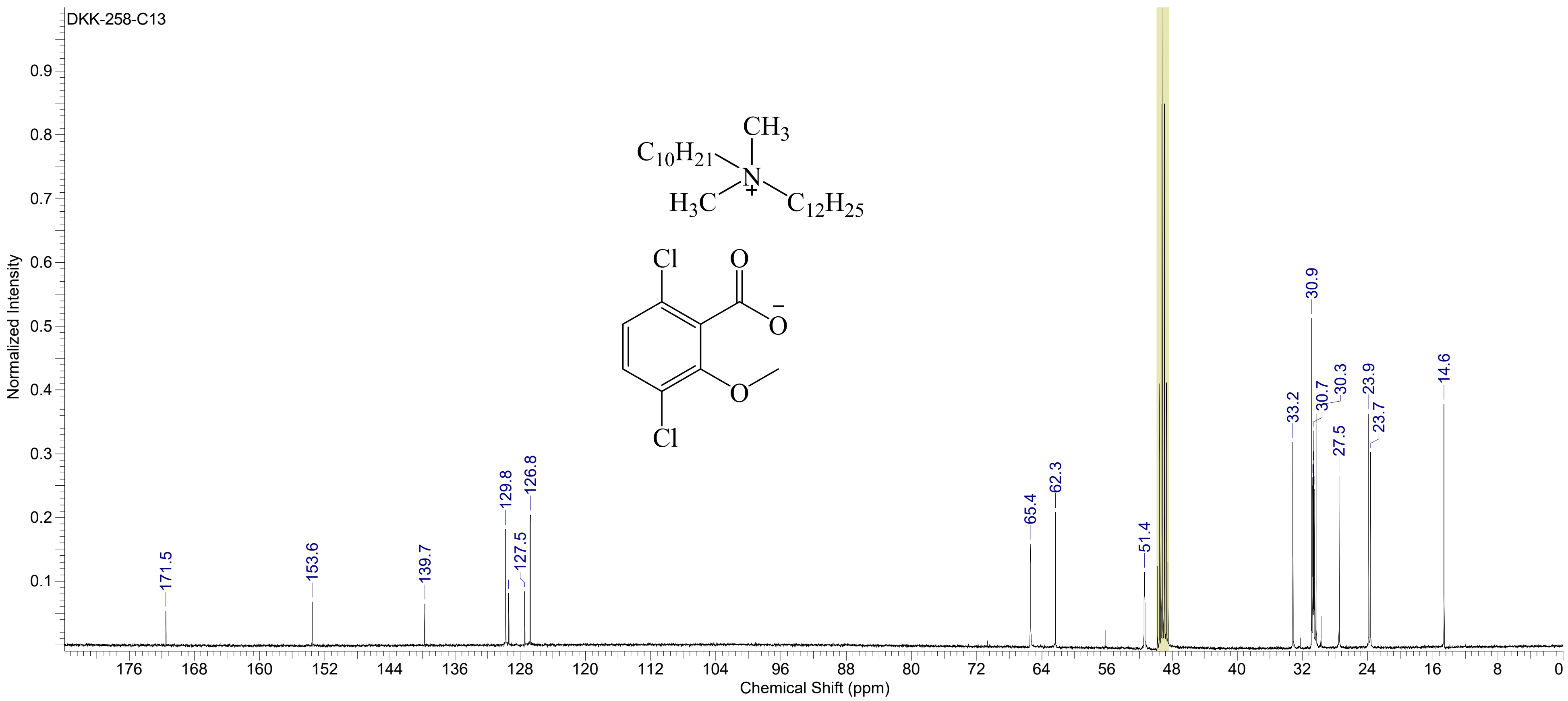

δC (METHANOL-d4): 171.5, 153.6, 139.7, 129.8, 129.4, 127.5, 126.8, 65.4 (2C), 62.3, 51.4 (2C), 33.2 (2C), 30.9 (2C), 30.7 (2C), 30.6 (4C), 30.3 (2C), 27.5 (2C), 23.9 (2C), 23.7 (2C), 14.6 (2C).

Figure S6. ^{13}C NMR spectrum of dodecyldecyldimethylammonium (3,6-dichloro-2-methoxy)benzoate (**3**)

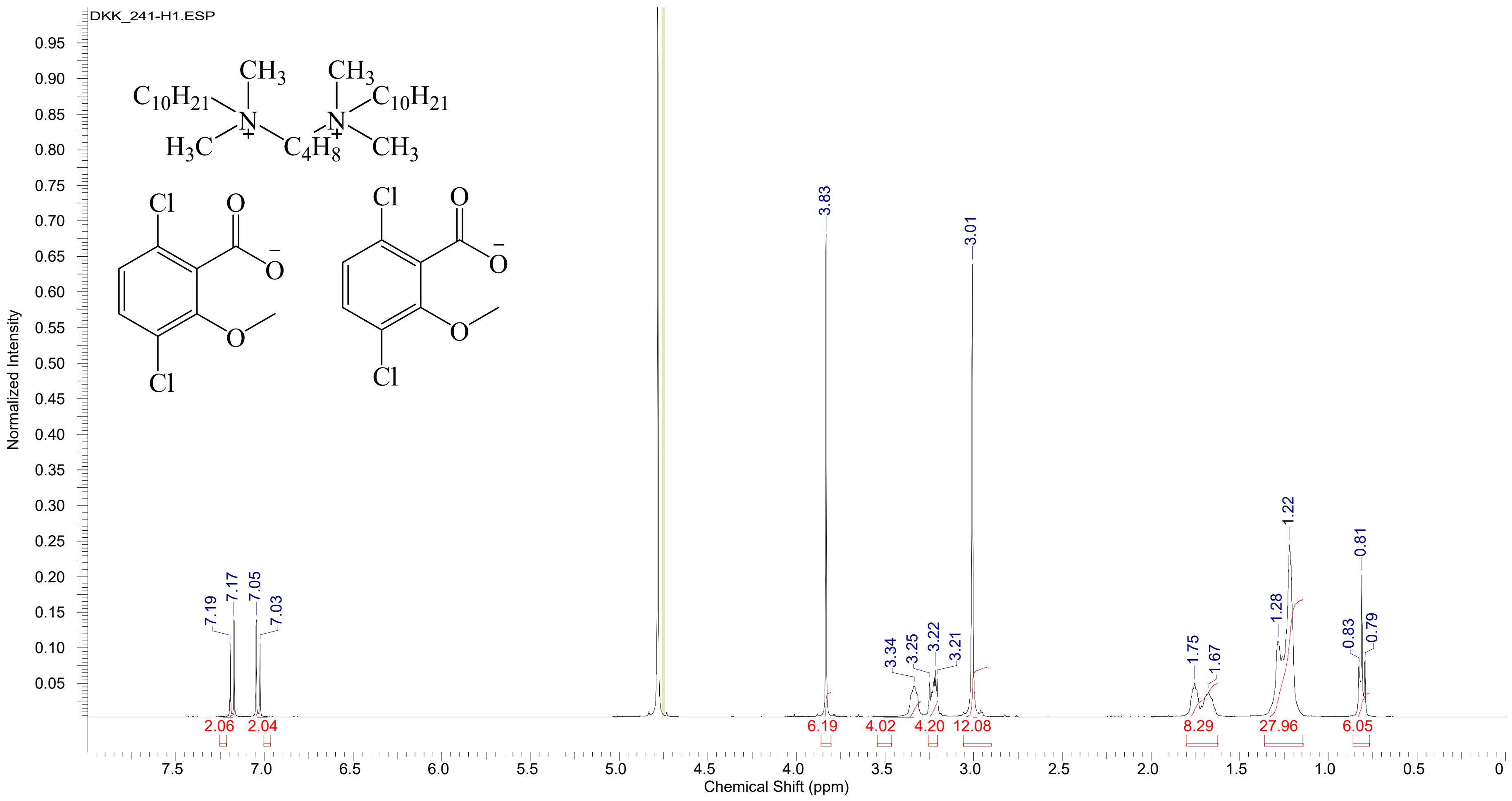

δ_H (METHANOL-d_4): 7.19 (2H, d, *J*=9 Hz), 7.05 (2H, d, *J*=9 Hz), 3.83 (6H, s), 3.34 (4H, br. s.), 3.21 - 3.25 (4H, m), 3.01 (12H, s), 1.67 - 1.75 (8H, m), 1.22 - 1.28 (28H, m), 0.79 - 0.83 (6H, m).

Figure S7. ^{1}H NMR spectrum of tetramethylene-1,4-(decyldimethylammonium) di[(3,6-dichloro-2-methoxy)benzoate] (**4**)

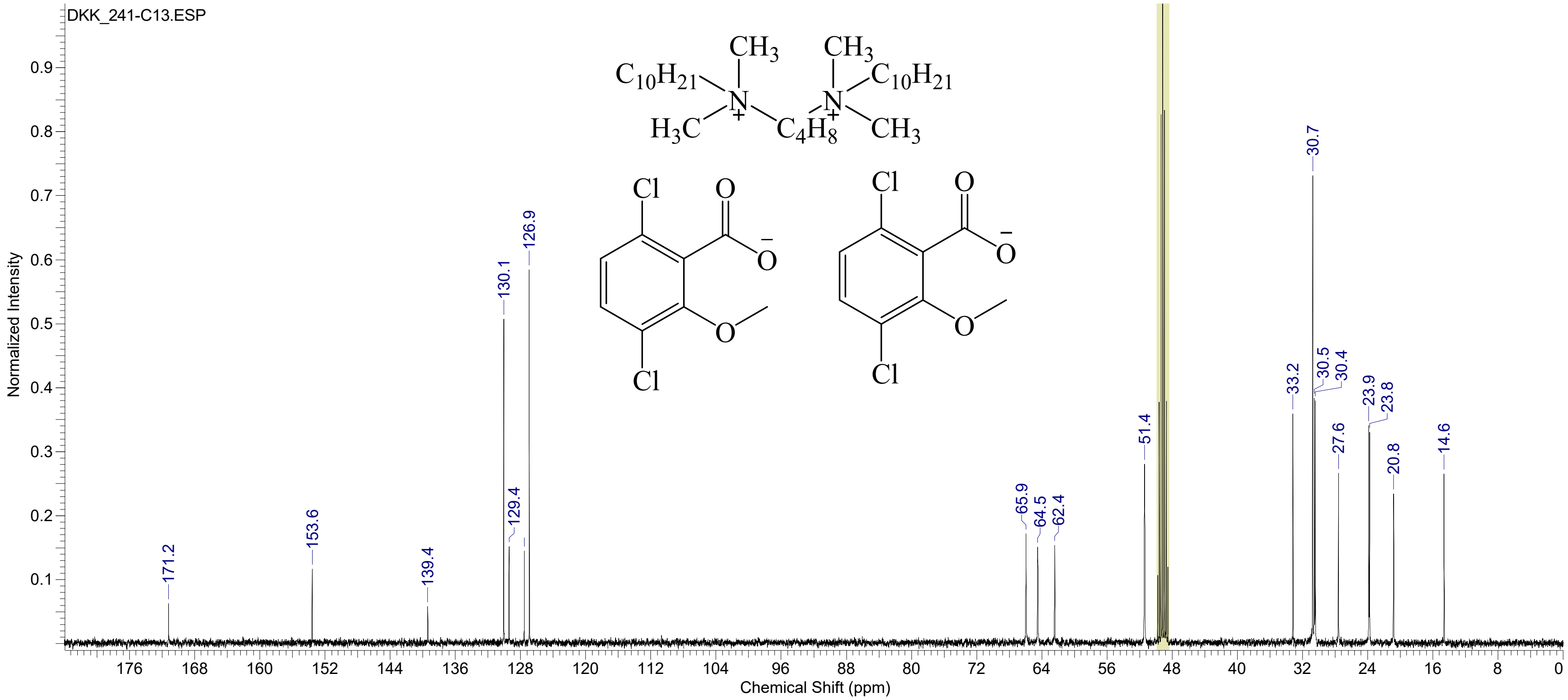

δC (METHANOL-d4): 171.2 (2C), 153.6 (2C), 139.4 (2C), 130.1 (2C), 129.4 (2C), 127.5 (2C), 126.9 (2C), 65.9 (2C), 64.5 (2C), 62.4 (2C), 51.4 (4C), 33.2 (2C), 30.7 (2C), 30.5 (2C), 30.4 (4C), 27.6 (2C), 23.9 (2C), 23.8 (2C), 20.8 (2C), 14.6 (2C).

Figure S8. ^{13}C NMR spectrum of tetramethylene-1,4-(decyldimethylammonium) di[(3,6-dichloro-2-methoxy)benzoate] (**4**)

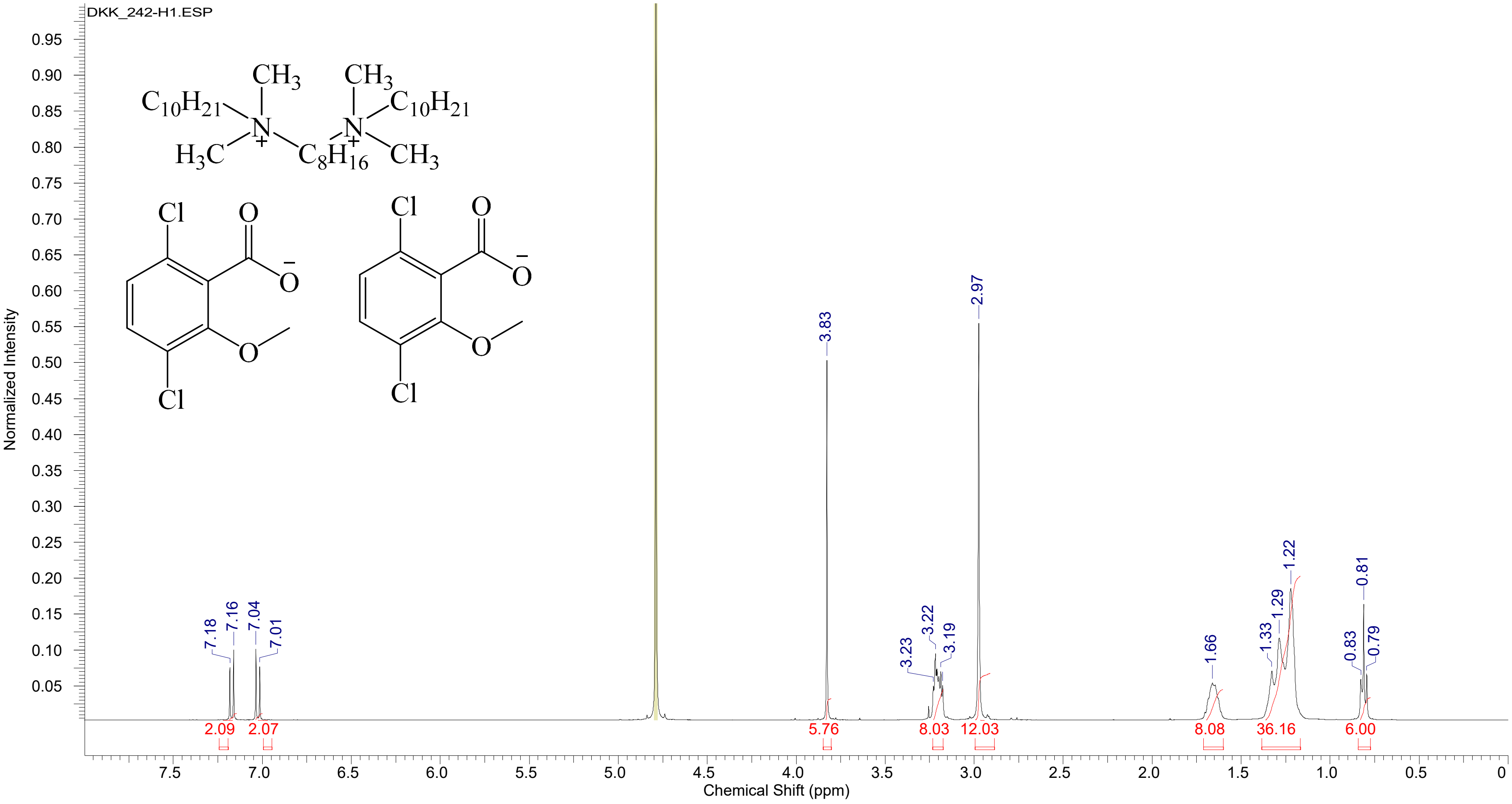

δH (METHANOL-d4): 7.18 (2H, d, *J*=9 Hz), 7.04 (2H, d, *J*=9 Hz), 3.83 (6H, s), 3.19 - 3.23 (8H, m), 2.97 (12H, s), 1.66 (8H, m), 1.22 - 1.33 (36H, m), 0.79 - 0.83 (6H, m).

Figure S9. ^{1}H NMR spectrum of octamethylene-1,8-bis(decyldimethylammonium) di[(3,6-dichloro-2-methoxy)benzoate] (**5**)

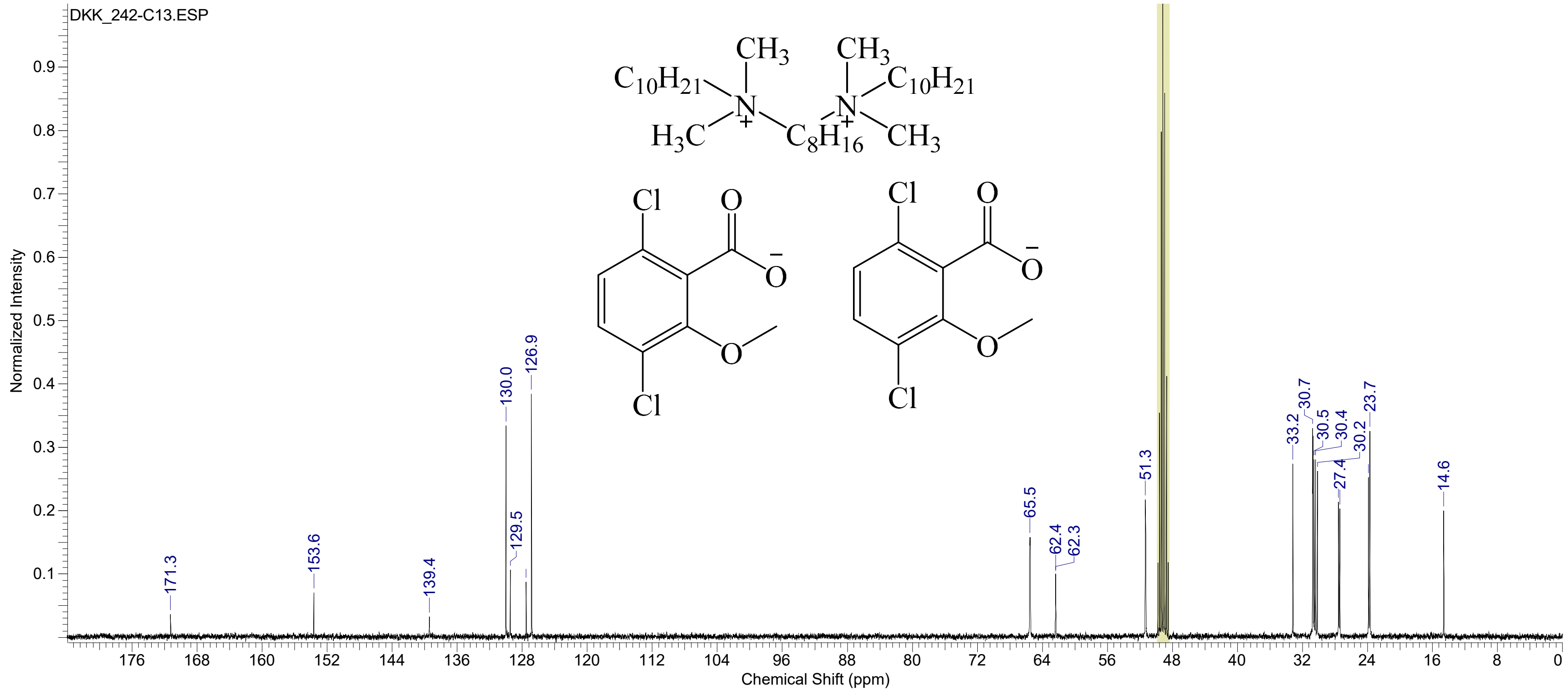

δc (METHANOL-d4): 171.3 (2C), 153.6 (2C), 139.4 (2C), 130.0 (2C), 129.5 (2C), 127.5 (2C), 126.9 (2C), 65.5 (2C), 62.4 (2C), 62.3 (2C), 51.3 (4C), 33.2 (2C), 30.7 (2C), 30.5 (2C), 30.4 (2C), 30.2 (4C), 27.5 (2C), 27.4 (2C), 23.9 (4C), 23.7 (2C), 14.6 (2C).

Figure S10. ^{13}C NMR spectrum of octamethylene-1,8-bis(decyldimethylammonium) di[(3,6-dichloro-2-methoxy)benzoate] (**5**)

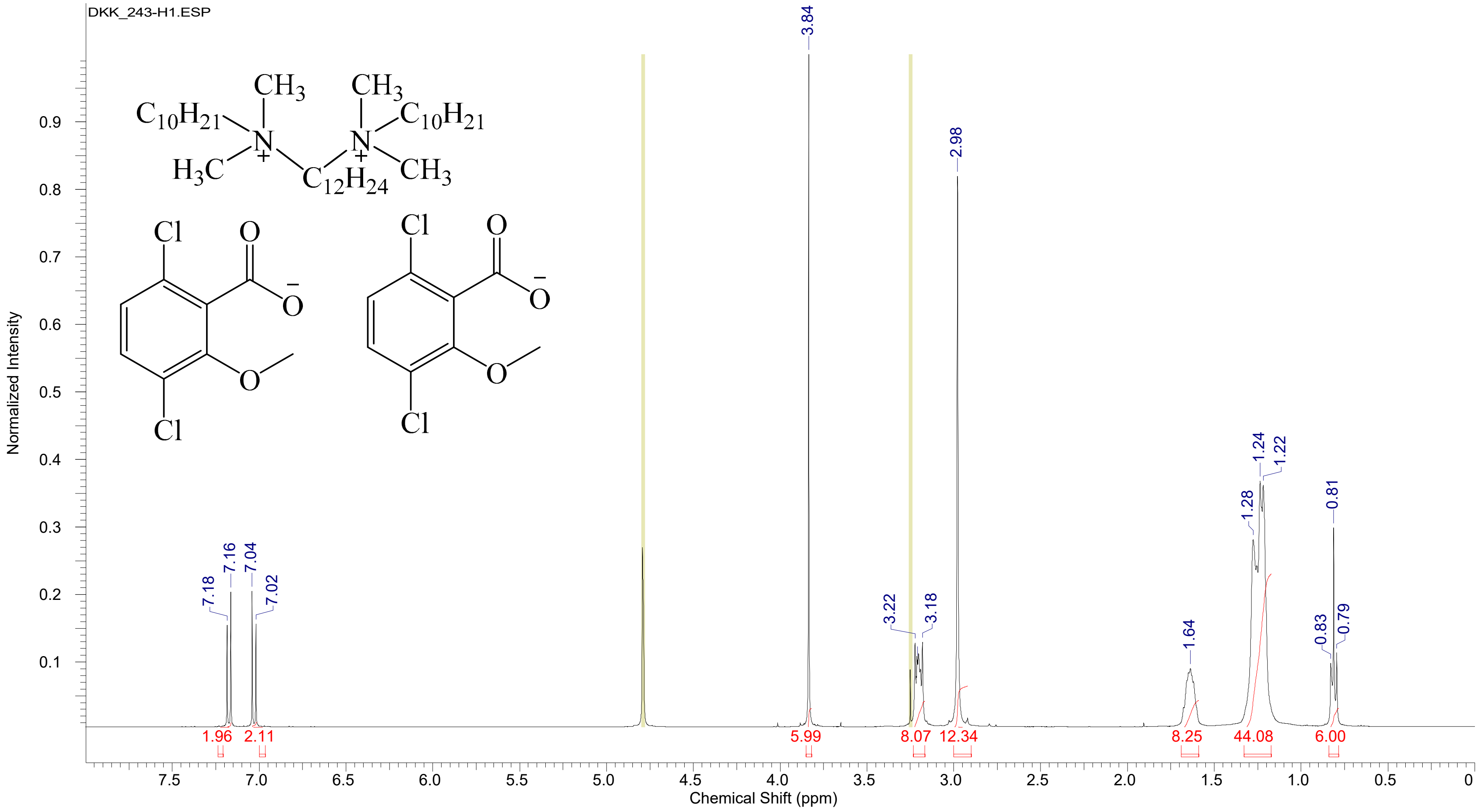

δH (METHANOL-d4): 7.18 (2H, d, *J*=9 Hz), 7.04 (2H, d, *J*=9 Hz), 3.84 (6H, s), 3.18 - 3.22 (8H, m), 2.98 (12H, s), 1.64 (8H, m), 1.22 - 1.28 (44H, m), 0.79 - 0.83 (6H, m).

Figure S11. ^{1}H NMR spectrum of dodecamethylene-1,12-bis(decyldimethylammonium) di[(3,6-dichloro-2-methoxy)benzoate] (**6**)

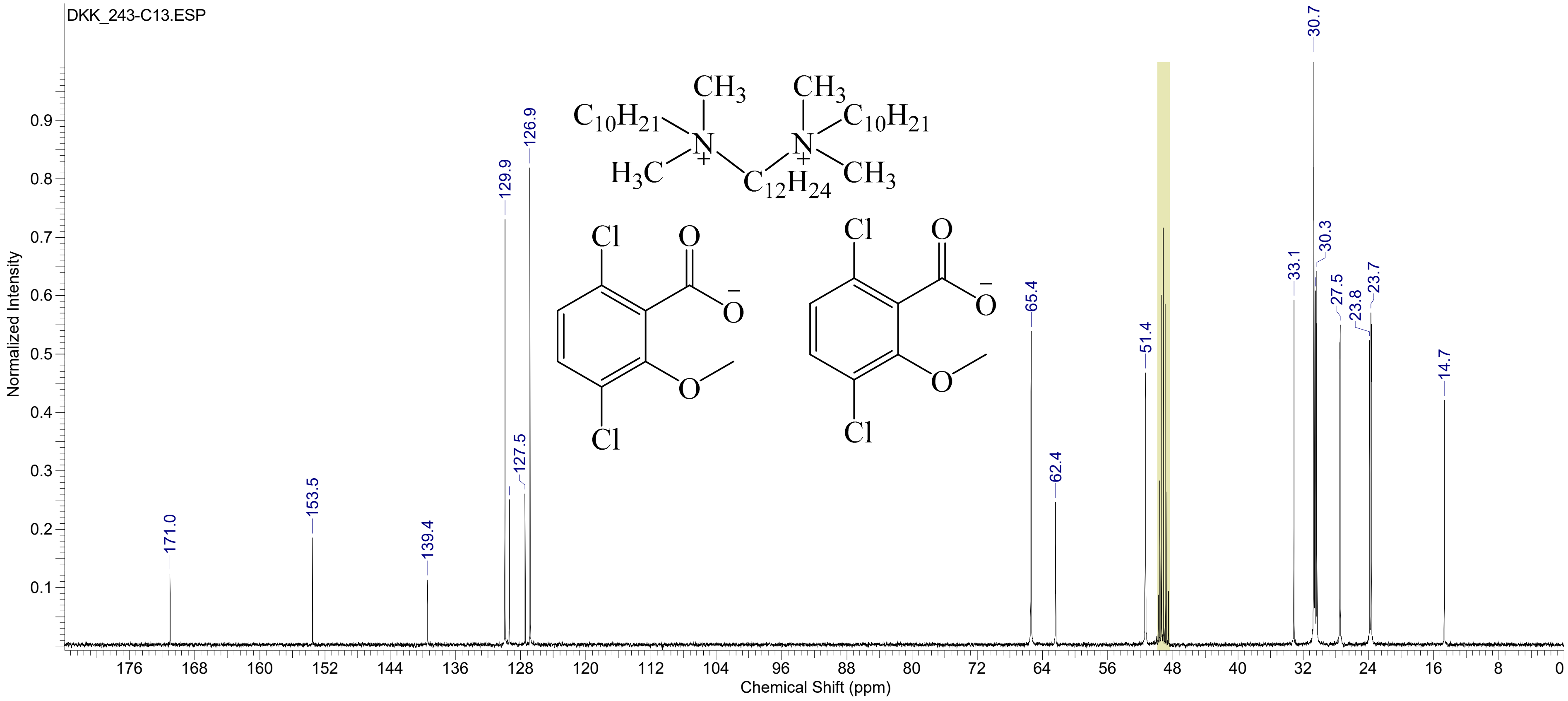

δC (METHANOL-d4): 171.0 (2C), 153.5 (2C), 139.4 (2C), 129.9 (2C), 129.4 (2C), 127.5 (2C), 126.9 (2C), 65.4 (4C), 62.4 (2C), 51.4 (4C), 33.1 (2C), 30.7 (4C), 30.5 (4C), 30.3 (2C), 30.3 (4C), 27.5 (4C), 23.8 (4C), 23.7 (2C), 14.6 (2C).

Figure S12. ^{13}C NMR spectrum of dodecamethylene-1,12-bis(decyldimethylammonium) di[(3,6-dichloro-2-methoxy)benzoate] (**6**)

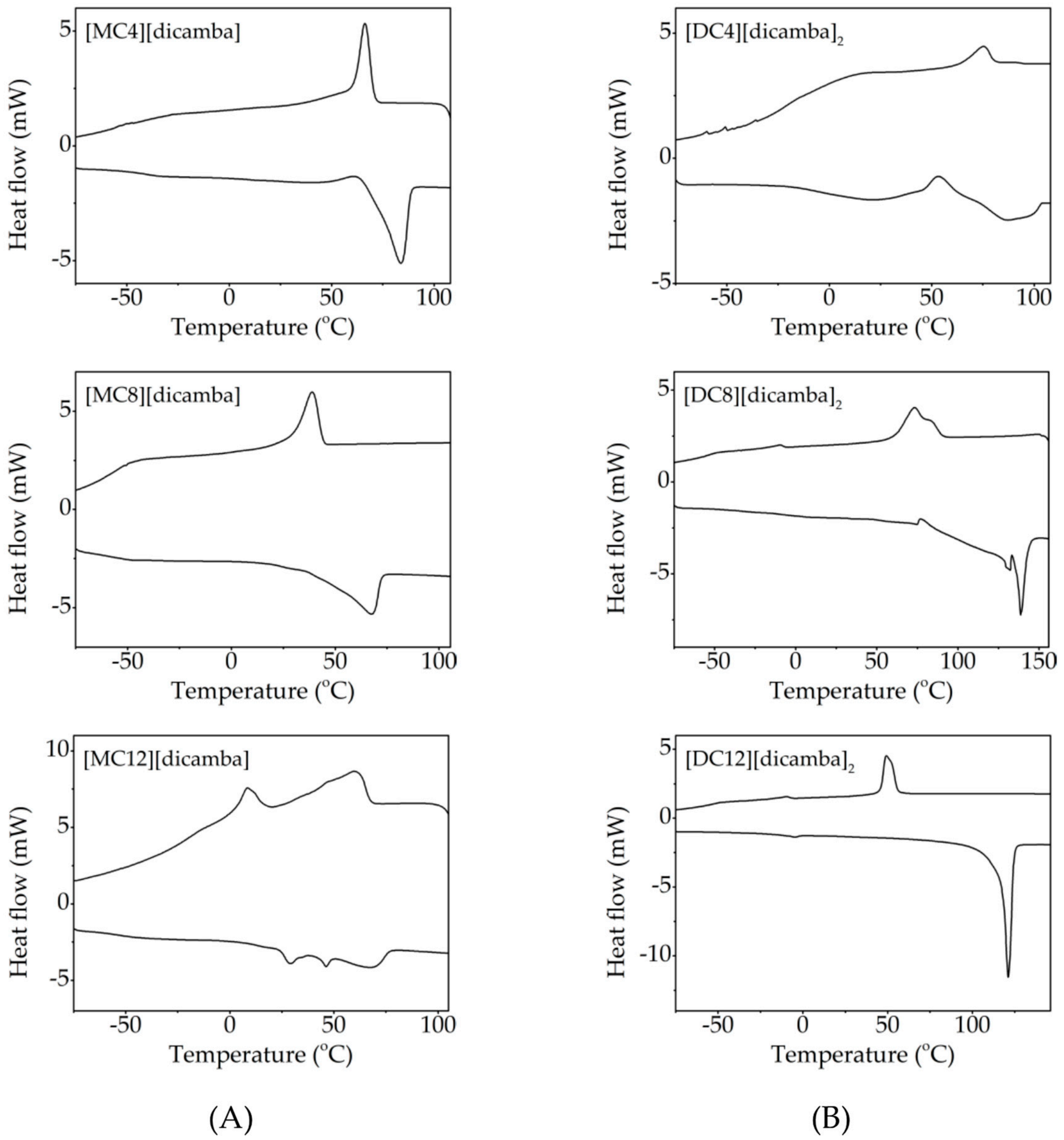

Figure S13. DSC thermograms of the monocationic (A) and dicationic (B) compounds with (3,6 dichloro-2-methoxy)benzoate anion

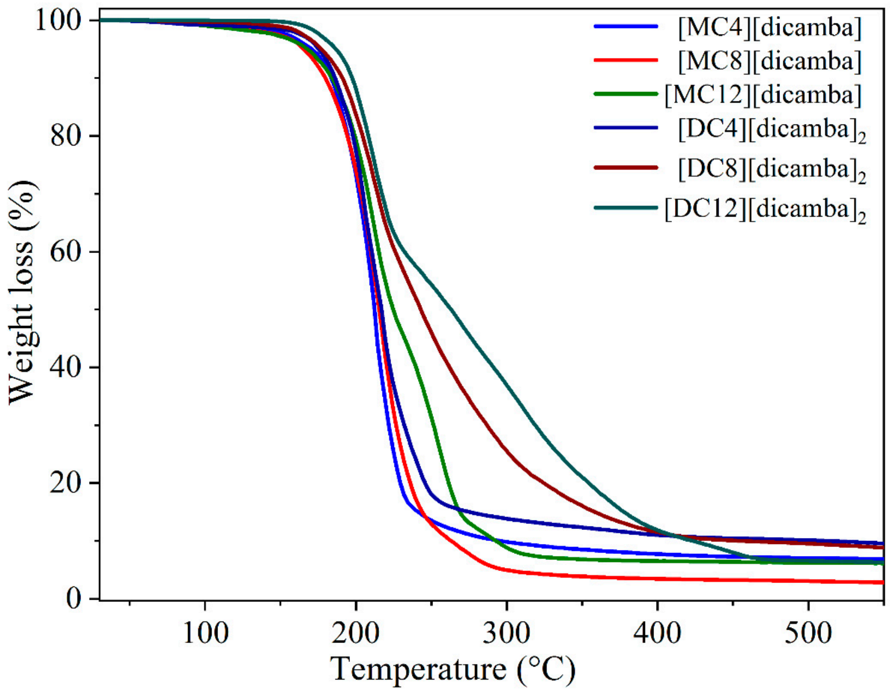

(A)

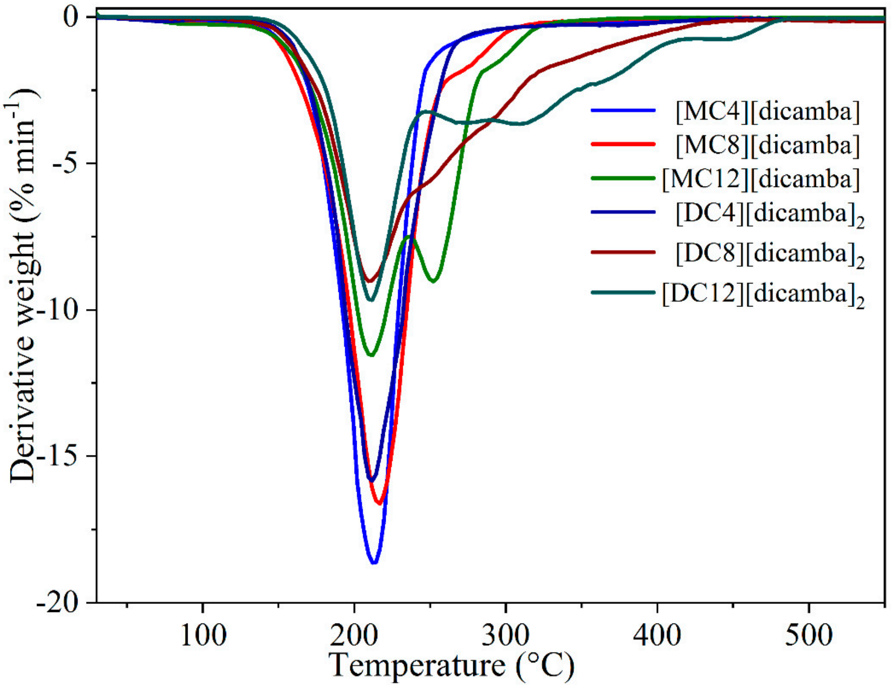

(B)

Figure S14. (A) TG and (B) DTG curves for investigated compounds

Table S1. Contact angle values of compounds (biological surface)

Abbreviation	WINTER RAPESEED CA (°)		
	5 (°C)	15 (°C)	25 (°C)
**[MC4][dicamba]**	63.32	57.67	57.32
**[MC8][dicamba]**	54.23	52.30	49.74
**[MC12][dicamba]**	50.69	47.50	46.64
**[DC4][dicamba]2**	60.32	56.67	52.32
**[DC8][dicamba]2**	52.10	50.34	47.12
**[DC12][dicamba]2**	51.64	44.54	42.23

Abbreviation	COMMON WHEAT CA (°)		
	5 (°C)	15 (°C)	25 (°C)
**[MC4][dicamba]**	83.43	82.00	80.00
**[MC8][dicamba]**	79.21	77.21	76.21
**[MC12][dicamba]**	65.34	63.34	61.34
**[DC4][dicamba]2**	78.43	83.12	74.34
**[DC8][dicamba]2**	72.34	75.24	70.76
**[DC12][dicamba]2**	64.32	68.23	60.65

Abbreviation	CORNFLOWER CA (°)		
	5 (°C)	15 (°C)	25 (°C)
**[MC4][dicamba]**	65.85	56.67	52.32
**[MC8][dicamba]**	59.84	54.25	49.74
**[MC12][dicamba]**	45.28	42.26	40.64
**[DC4][dicamba]2**	60.76	51.98	48.11
**[DC8][dicamba]2**	56.98	47.76	46.23
**[DC12][dicamba]2**	40.12	35.65	39.12

Abbreviation	WHITE MUSTARD CA (°)		
	5 (°C)	15 (°C)	25 (°C)
**[MC4][dicamba]**	62.76	59.23	54.77
**[MC8][dicamba]**	55.23	52.02	45.08
**[MC12][dicamba]**	49.99	49.02	41.85
**[DC4][dicamba]2**	58.76	56.23	51.87
**[DC8][dicamba]2**	48.23	49.12	42.08
**[DC12][dicamba]2**	45.12	44.12	38.65

Table S2. Contact angle values of compounds (membrane)

Abbreviation	CA (°)
**[MC4][dicamba]**	44.00
**[MC8][dicamba]**	37.02
**[MC12][dicamba]**	36.18
**[DC4][dicamba]2**	42.39
**[DC8][dicamba]2**	38.28
**[DC12][dicamba]2**	38.21

Equations necessary to determine parameters related to surface activity.

Gibbs energy (ΔG^0_{ads}) which characterizes the interactions between the bulk phase and the surface phase:

$$\Delta G^0{}_{ads} = -RTlna$$

where:

R is gas constant,
T is absolute temperature.

The a value is a parameter of the Szyszkowski equation:

$$\gamma = \gamma_0[1 - b\ln\left(\frac{c}{a} + 1\right)]$$

where:

$\gamma 0$ is the surface tension of the solvent.

Surface excess concentrations (Γ_{max}) were calculated from the slope of the linear portion of the γ-log C plots (Fig. 4) using the Gibbs isotherm:

$$\Gamma_{max} = -\frac{1}{RT} \cdot \frac{d\gamma}{d(\ln C)}$$

where:

R is gas constant,
T is absolute temperature,
C is concentration of salts.

From Γ_{max} the minimum surface occupied by a molecule at the interface A_{min} can be calculated from equation:

$$A_{min} = \frac{1}{\Gamma_{max} N_A}$$

where:

N_A is the Avogadro number.

Surface pressure at the CMC (Π_{CMC}) can be calculated from equation:

$$\pi_{CMC} = \gamma_0 - \gamma_{CMC}$$

where:

γ_0 is surface tension of pure water which equals 72.8 mN m^{-1} (at under measurement conditions).

Adsorption efficiency (pC_{20}):

$$pC_{20} = -\log C_{20}$$
